# Diploid genomic architecture of *Nitzschia inconspicua*, an elite biomass production diatom

**DOI:** 10.1038/s41598-021-95106-3

**Published:** 2021-08-02

**Authors:** Aaron Oliver, Sheila Podell, Agnieszka Pinowska, Jesse C. Traller, Sarah R. Smith, Ryan McClure, Alex Beliaev, Pavlo Bohutskyi, Eric A. Hill, Ariel Rabines, Hong Zheng, Lisa Zeigler Allen, Alan Kuo, Igor V. Grigoriev, Andrew E. Allen, David Hazlebeck, Eric E. Allen

**Affiliations:** 1 Center for Marine Biotechnology and Biomedicine, Scripps Institution of Oceanography, University of California, San Diego, La Jolla, CA USA; 2 Global Algae Innovations, Lihue, HI USA; 3 Microbial and Environmental Genomics Group, J. Craig Venter Institute, La Jolla, CA USA; 4 Biological Sciences Division, Pacific Northwest National Laboratory, Richland, WA USA; 5 U.S. Department of Energy Joint Genome Institute, Lawrence Berkeley National Laboratory, Berkeley, USA; 6 Department of Plant and Microbial Biology, University of California Berkeley, Berkeley, CA USA; 7 Center for Microbiome Innovation, University of California, San Diego, La Jolla, CA USA; 8 Division of Biological Sciences, University of California, San Diego, La Jolla, CA USA

**Keywords:** Plant biotechnology, Genomics, Comparative genomics, Plant sciences

## Abstract

A near-complete diploid nuclear genome and accompanying circular mitochondrial and chloroplast genomes have been assembled from the elite commercial diatom species *Nitzschia inconspicua*. The 50 Mbp haploid size of the nuclear genome is nearly double that of model diatom *Phaeodactylum tricornutum*, but 30% smaller than closer relative *Fragilariopsis cylindrus*. Diploid assembly, which was facilitated by low levels of allelic heterozygosity (2.7%), included 14 candidate chromosome pairs composed of long, syntenic contigs, covering 93% of the total assembly. Telomeric ends were capped with an unusual 12-mer, G-rich, degenerate repeat sequence. Predicted proteins were highly enriched in strain-specific marker domains associated with cell-surface adhesion, biofilm formation, and raphe system gliding motility. Expanded species-specific families of carbonic anhydrases suggest potential enhancement of carbon concentration efficiency, and duplicated glycolysis and fatty acid synthesis pathways across cytosolic and organellar compartments may enhance peak metabolic output, contributing to competitive success over other organisms in mixed cultures. The *N. inconspicua* genome delivers a robust new reference for future functional and transcriptomic studies to illuminate the physiology of benthic pennate diatoms and harness their unique adaptations to support commercial algae biomass and bioproduct production.

## Introduction

*Nitzschia* is a globally distributed genus of pennate diatoms found in benthic and planktonic habitats from both freshwater and marine environments. *Nitzschia* species have been identified as especially promising candidates for production of algae-derived biofuels and bioproducts via large-scale aquaculture, due to their prolific biomass production, high intracellular lipid content, and robust growth characteristics over a wide range of environmental conditions^1,2^. However, molecular optimization of photosynthetic *Nitzschia* candidate strains for large-scale commercial operations suffers from a lack of genomic sequence data, hindering the effective application of genetic engineering tools and interpretation of experimentally generated gene and protein expression data. Not knowing the extent to which desirable characteristics might be encoded by homozygous or heterozygous alleles also makes it difficult to predict the potential stability of these traits to recombination during sexual reproduction and selective breeding.

Although diatom nuclear genomes are generally obtained from diploid vegetative cells rather than haploid gametes, historical sequencing and assembly technologies often collapse closely related allelic duplicates into single consensus sequences. Aside from well-characterized model organisms like *Thalassiosira pseudonana*^3^ and *Phaeodactylum tricornutum*^4^, most currently available diatom genomes contain hundreds to thousands of small contigs that cannot be assigned to individual chromosomes^5–9^. These short fragments can be difficult to distinguish from sequencing and assembly errors, requiring extensive transcriptome data for validation^10^ and potentially leading to omission of genuine allelic variants.

Recently developed long read sequencing technologies coupled with modern, diploid-aware assembly programs provide an opportunity to address many of these issues, including enhanced abilities to resolve diploid alleles and low complexity repeat elements^11,12^. Here we present the first photosynthetic genome representative from genus *Nitzschia*. This near-complete diploid nuclear genome and accompanying organelle assemblies provide new insights into the genomic capabilities of pennate diatoms in the context of allelic diversity, and robust new references for future functional and transcriptomic studies.

## Results

### Cellular description and taxonomy

**Figure 1:**
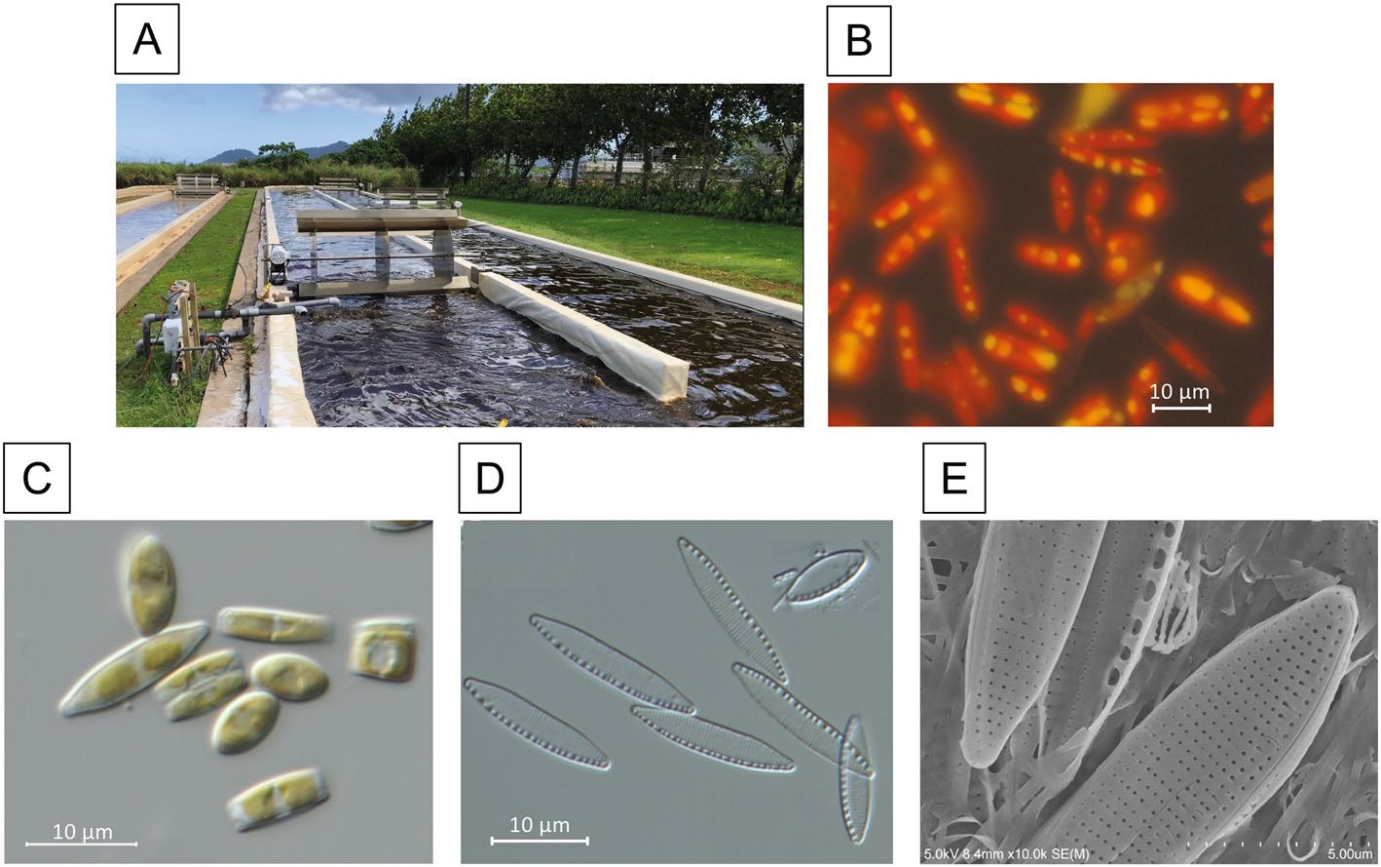
Outdoor culture and cellular morphology of *N. inconspicua* str. hildebrandi. Panel **A**, *N. inconspicua* cultivated in a paddle wheel pond at the Kauai Algae Farm operated by Global Algae Innovations Inc; **B**, fluorescence micrograph of cells stained with Nile Red, showing non-polar lipid droplets (yellow) and chlorophyll autofluorescence (red); **C**, differential interference contrast micrograph (DIC) of live cells in valve view and girdle view, showing elongated chloroplasts, small lipid droplets, and centrally located nucleus; **D**, DIC micrograph of acid cleaned valves showing lanceolate frustule shape and arrangement of striae and fibulae; **E**, scanning electron micrograph showing structural details of striae and fibulae.

*Nitzschia* diatom isolate GAI-293, originally collected from the tidal area of a stream near Lihue on the island of Kauai, Hawaii, USA, was isolated in axenic culture by Global Algae Innovations. This strain is euryhaline and can be cultivated in sea water and low salinity brackish water, matching the ecological description of *N. inconspicua*^13^. Isolate GAI-293 is capable of growth using a CO_2_ supply derived from power plant flue gas and using recycled growth media in large-scale outdoor raceways (Fig. 1A), demonstrating average productivity of 22 g/m^2^/day on an ash-free dry weight basis over a 2.5 month cultivation period. Vegetative cells have a typical raphid pennate morphology, with gliding motility and prominent lipid droplets visible under conditions of silica depletion in the media (Fig. 1B). Auxospores are formed paedogamously following a fusion of gametes produced within the same gametangium^14^.

Species determinations based on cellular morphology can be difficult within genus *Nitzschia*^15^, but cell shape (lanceolate), length (5.7–24.9 µm) width (3.0–4.3 µm), and numbers of striae (24–29) and fibulae (10–15) per 10 µm for laboratory cultures of isolate GAI-293 (Figs. 1C–F) were consistent with previous literature descriptions of *Nitzschia inconspicua*^13,15,16^. Longer vegetative cells (> 33 µm) were sometimes observed in live material from outdoor cultivation, and the largest initial cell observed post-auxospore formation was 46 µm long.

**Figure 2:**
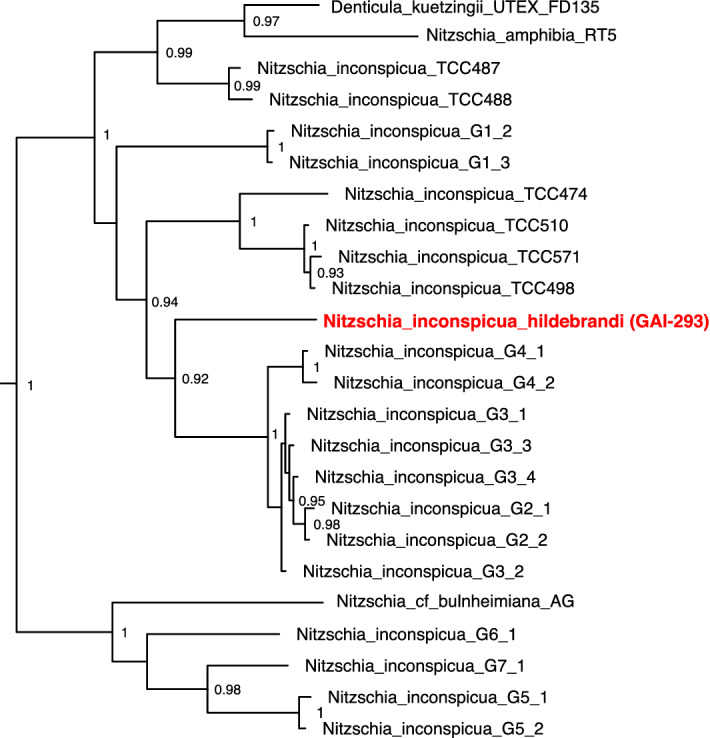
Concatenated multilocus gene tree of *Nitzschia inconspicua* strains. Alignments were constructed from 18S (SSU) and 23S (LSU) nuclear rRNA genes combined with chloroplast genes psbC (photosystem II reaction center protein C), and rbcL (ribulose bisphosphate carboxylase large chain). Supplementary Figure S1 shows a more complete version of this tree including the entire Bacillariaceae family. Accession numbers for concatentated genes are provided in Supplementary Table S5.

Molecular taxonomic classification was confirmed using a concatenated 4-gene multilocus tree, constructed from 18S and 23S nuclear rRNA genes combined with chloroplast genes *psbC* (photosystem II reaction center protein C), and *rbcL* (ribulose bisphosphate carboxylase large chain). Isolate GAI-293 formed a well-supported, independent branch nested within the *N. inconspicua* clade of the Bacillariaceae family (Fig. 2, Supplementary Figure S1)^17^. To honor the memory of diatom biologist Dr. Mark Hildebrand (1958–2018), who worked on lipid formation in this diatom and was a strong supporter of diatoms for biofuels and bioproducts, this organism has been given the name *Nitzschia inconspicua* strain hildebrandi.

### Genome sequencing and assembly

DNA was extracted from actively growing, diploid vegetative cells and used to generate 646,344 PacBio reads, ranging in size from 1,000 to 117,697 nt, with an average length of 31,140 nt. Long reads were assembled using the diploid-aware program CANU, producing 123 ungapped, nuclear contigs plus individual circular contigs for mitochondrial and chloroplast genomes (Table 1). Mitochondrial and chloroplast contigs were recognizable by their divergent nucleotide compositions of 29.3% and 33.4% G + C, respectively, versus an average of 45.1% ± 0.9% for nuclear sequences.

**Table 1:** Genomic properties of *Nitzschia inconspicua* str. hildebrandi and related diatoms. Genome size and number of predicted proteins shown for *N. inconspicua* are for the entire diploid assembly, with estimated haploid values in parentheses.

	*Nitzschia inconspicua* str. hildebrandi	*Pseudo-nitzschia multistriata B856*	*Fragilariopsis cylindrus CCMP1102*	*Phaeodactylum tricornutum v2.0*	*Fistulifera solaris JPCC DA0580*	*Semanivis robusta D6*	*Thalassiosira pseudonana v3.0*
Nuclear genome size (Mbp)	99.7 (49.9)	56.8	80.5	26.1	49.7	125.6	31.3
Num nuclear contigs	123	700	271	33	295	4,754	24
Max ctg length	6,574,884	679,566	5,926,375	2,535,400	904,706	318,117	3,042,585
N50	3.7 Mbp	141 Kbp	782 Kbp	423 Kbp	330 Kbp	51 Kbp	1.3 Mbp
GC content (%)	45.1	43.3	39.1	50.6	45.6	48.3	47.8
% coding sequences	59%	34%	28%	54%	61%	77%	50%
Predicted proteins	38,601 (17,968)	12,039	18,111	10,402	20,429	36,254	11,766
BUSCO completeness	100%	86%	95%	94%	97%	99%	94%
% CDS complete proteins (start/stop codons)	100%	99%	87%	84%	100%	98%	80%
Plastid genome (bp)	139,309	nd	123,275	117,369	134,918	150,240	128,813
Mitochondrial genome (bp)	69,563	nd	nd	77,356	39,648	44,018	43,827
Morphology	pennate	pennate	pennate	pennate	pennate	pennate	centric
Genome Sequencing technology	PacBio	Sanger	Sanger	Sanger	454	Illumina + PacBio	Sanger

The *N. inconspicua* haploid protein number is less than half of the diploid value because it was calculated using historical conventions typically applied to older diatom genomes, excluding allelic duplicates, alternative transcripts, and low complexity regions, as described in Materials and Methods. Additional information on BUSCO completeness scores is presented in Supplementary Table 2.

Overall quality of the *N. inconspicua* assembly is higher than most currently available diatom genomes, based on sizes and numbers of contigs, completeness of conserved BUSCO marker genes, and percentage of non-fragmented protein predictions, defined as coding sequences that begin and end with canonical start and stop codons (Table 1). Six assembled contigs, ranging in size from 2.2–4.1 Mbp, were capped with telomeres of 355–414 nt at both 5' and 3' ends, suggesting complete chromosomes. Exceptional assembly quality was made possible by high-coverage, long-read PacBio read technology and the ability of the CANU assembler to resolve allelic ambiguities^18–20^.

**Figure 3:**
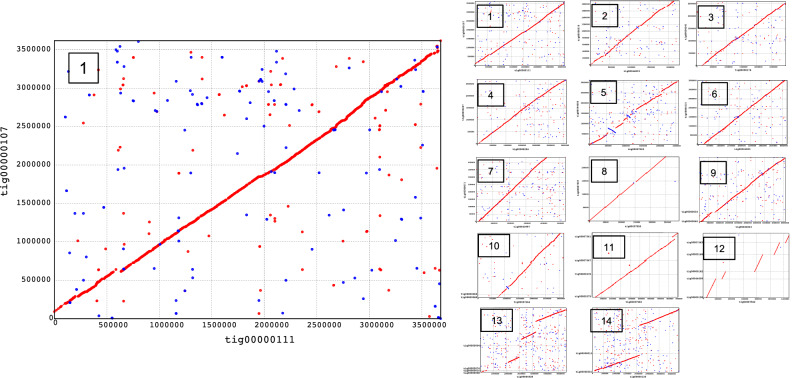
MUMmer dot plots of syntenic contigs in *N. inconspicua* diploid assembly. Enlarged view of pair #1 shows nucleotide positions for contigs along the x and y axes, with colored dots and lines indicating match direction (red = forward, blue = reverse). Full sized images for all 14 thumbnail views in are presented in Supplementary Figure S2. Assembly properties of paired contigs are described in Supplementary Table S1.

The 99.7 Mbp size of the nuclear assembly and its 38,601 predicted proteins were larger than expected based on previously published numbers for other diatom species. However, diatom genome size statistics are conventionally reported as haploid values, even when obtained from diploid organisms, making an estimated haploid size of 49.9 Mbp and 17,988 predicted proteins for *N. inconspicua* unremarkable. Assembly diploidy was first demonstrated by MUMmer dot plots of paired contigs (Fig. 3, Supplementary Figures S2A-S2M). 42 contigs were resolved into 14 highly syntenic paired alignments, at approximately 94% average nucleotide identity per pair (including introns and non-coding, as well as coding sequences) and nearly equal assembly coverage depths between partner contigs (Supplementary Table S1). Paired contig alignments account for 93% (92,897,432 nt) of the nuclear assembly. At least eleven of the matched contig sets have few insertions, deletions, or re-arrangements, but it is not clear whether incompleteness of the remaining alignments, covering multiple contigs, are due to biologically relevant sequence differences, assembly errors, or some combination of these factors.

Additional evidence supporting diploid completeness of the assembly was obtained using predicted protein sequences based on BRAKER2^21^ gene models. Not only were 100% of the conserved BUSCO markers from Stramenopiles present in predicted *N. inconspicua* gene models, but 92% of these markers were also present in a second, unfragmented copy, consistent with the 92.8% genome alignment coverage observed in MUMmer matched contig pairs (Supplementary Table S2). Duplicated BUSCO gene markers were not observed in any other previously published diatom genome at greater than 1%, except for putatively alloploid *Fistulifera solaris*^6^.

Diploid assembly and gene model correctness were further validated by comparing coding sequences for all 38,601 predicted proteins with 452,784 transcriptome sequences that were generated from growth under a variety of environmental conditions and de novo assembled (Supplementary Table S3). 98.8% of the coding sequences matched assembled transcriptome sequences at an average of 98.5% ± 2.5% nt identity (median 99.4%) (Supplementary Figure S3). These results are consistent with expression of both diploid alleles, supporting previous reports suggesting the absence of genome-wide silencing mechanisms in diatoms^2,22,23^.

To determine the level of genomic variability between diploid alleles, predicted coding sequences and corresponding translations were compared to each other to identify matched pairs (Supplementary Figure S4). Average nucleotide sequence identity for paired coding sequences was 97.3 ± 2.9%. 98% of the sequences were more than 90% identical to their partners, and 32% were 100% identical. These results are consistent with an independent estimate of 3.5% overall genome heterozygosity, obtained from unassembled raw reads using relative k-mer abundance (Supplementary Figure S5).

### Non-protein coding genes

The *N. inconspicua* genome contained multiple copies of large and small subunit rRNAs and tRNAs for all 20 standard amino acids plus tRNA-SeC, potentially enabling the incorporation of selenocysteine. Paired allelic copies of other well-known RNA genes were also present, including a THI element TPP riboswitch (thiamine biosynthesis regulator), a 5' *ureB* small RNA (urease regulator), U1 and U6 spliceosomal RNAs, and small nucleolar RNAs of types SNORD24 and SNORA11, potentially acting as guides for the methylation of RNA targets and the conversion of uridine to pseudouridine, respectively.

**Figure 4:**
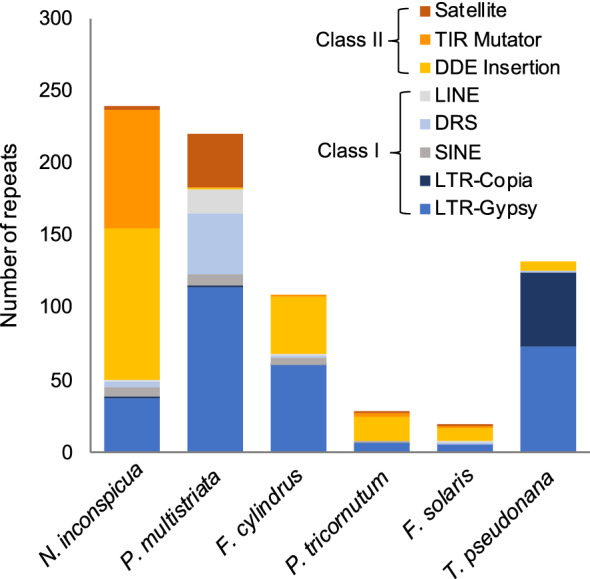
Repetitive element types in *N. inconspicua* versus other diatom genomes. Class designations refer to copy and paste (Class I) versus cut and paste (Class II) transposon replication mechanisms. Total numbers represent estimated haploid frequencies, so *N. inconspicua* totals have been divided by two.

Non-protein coding regions comprised 41% of the *N. inconspicua* nuclear genome, including 7% identified as low-complexity elements by RepeatModeler^24^. These relative proportions were similar to levels previously reported in finished diatom chromosomes from *P. tricornutum,* and *T. pseudonana*, but much lower than draft genomes for *P. multistriata*, and *F. cylindrus* (Table 1, percent coding sequences). *N. inconspicua* transposable elements were widely distributed across 44 different contigs (Fig. 4). The most abundant elements were Class II (cut and paste) sequences belonging to the DDE Insertion and Eukaryotic Terminal Interspersed Repeat Mutator (MULE) subtypes, followed by Class I (copy and paste) elements of the LTR-Gypsy subtype. Class II elements are rare in previously sequenced diatom genomes, and expansion of the MULE subtype is unique to *N. inconspicua*. Transposable elements were most abundant in contigs tig00044028, tig00000004, tig00000012 and tig00000120, potentially contributing to the lower alignment synteny shown in Fig. 3 for contig pairs 13 and 14.

The most frequently repeated sequence in the *N. inconspicua* nuclear genome (630 exact matches) was the 12-mer TTAGGGTTGGGG, a G-rich extension of the conserved eukaryotic telomere motif TTAGGG. The second half of this sequence (TTGGGG), originally described in the macronucleus of ciliate *Tetrahymena thermophila*^25,26^, is quite different from the consistent 6-mer telomer pattern used by *T. pseudonana* and *P. tricornutum* (TTAGGG), and the more T-rich sequences found in *Chlamydomonas reinhardtii* (TTTTAGGG) and most land plants (TTTAGGG)^27–30^. Tandem repeats containing multiple copies of *N. inconspicua* telomer motifs were found exclusively at the ends of contigs, including thirteen of the fourteen matched pair sets shown in Fig. 3.

*N. inconspicua* telomeres differ from those of *T. pseudonana* and *P. tricornutum,* whose 6-mer repeats are all identical, in containing additional degenerate, truncated subsequences of varying lengths interspersed between full-length 12-mers (Supplementary Figure S6). Although degenerate *N. inconspicua* telomer repeat patterns were supported by PacBio read assembly depths of 12 to 31-fold in multiple contigs (Supplementary Table S4), excluding all potential artifacts that might have been introduced during sequencing and assembly would require verification using an alternative technology (e.g. Illumina).

Examples of telomer pattern degeneracy are well-known in *Paramecium*^31^ and *Saccharomyces*^32^, but have not previously been reported in diatoms. Telomer sequence inconsistencies are believed to result from site specific nucleotide mis-incorporation in the telomerase enzyme of *Paramecium,* and slippage with premature termination of reverse transcriptase activity in *Saccharomyces*^33,34^. The variable lengths of partial repeats in *N. inconspicua* telomeres suggest greater consistency with the yeast degeneracy model. Amino acid sequences of the two *N. inconspicua* telomerase alleles are 95% identical to each other but only 37% identical to their closest diatom match (*P. multistriata*), consistent with potential differences from previously sequenced diatoms in enzyme processivity.

### Mitochondrial and chloroplast genomes

**Figure 5:**
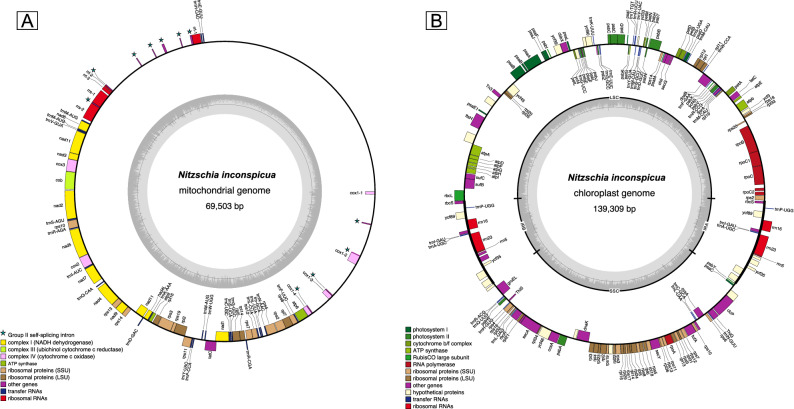
*N. inconspicua* organelle genome maps.

The circular *N. inconspicua* mitochondrial genome is larger than many other pennate diatoms due to a 19,055 nt intragenic spacer (Fig. 5A), consistent with the observation of intragenic spacers of widely varying sizes in mitochondrial genomes from other diatoms^35^. In addition to small and large mitochondrial rRNA subunits (rrnS, rrnL), the mitochondrial genome encodes ATP synthase complex components (atp 6 and 9), cytochrome B, cytochrome oxidase subunits (cox 1–3), NADH dehydrogenase subunits (nad 1–7, 9, and 11), large and small ribosomal subunit proteins (rpl 2, 5, 7, 14, and 16, plus rps 2, 3, 4, 7, 8, 10, 11, 13, 14, and 19), a tatC transporter, and 24 tRNA genes. COX1 and ribosomal subunit genes flanking the large intergenic spacer are interrupted by multiple group II introns matching RFAM patterns RF02012 and RF00029, but no sequences matching these motifs were detected within the spacer region itself. Group II introns have previously been reported to occur sporadically within COX1 and ribosomal rRNA genes in the mitochondrial sequences of other diatoms^35–38^. The large intergenic spacer region contained many low complexity repeats, but no coding sequences, RFAM database RNA gene matches, or blast matches to sequences in the Genbank nt database were detected.

The *N. inconspicua* chloroplast genome architecture is typical of that found in other diatoms, as well as many plants and algae, with two inverted repeats separating long single copy (LSC) and short single copy (SSC) regions (Fig. 5B). The inverted repeat sections are each 13,169 nt long and contain three ribosomal RNA genes and two tRNAs. Chloroplast genes encoded in non-duplicated regions include those associated with photosystem I, photosystem II, cytochrome b/f complex, ATP synthase, RuBisCO, thiamin synthesis, tRNAs, RNA polymerase, large and small subunit RNA proteins, TatC and SecA/SecY type transporters, molecular chaperones, antioxidants, and quality control protease FtsH. Five conserved open reading frames of unknown function (ycf39, ycf45, ycf66, ycf89, ycf90) were shared with the *F. cylindrus* chloroplast genome at 56–85% amino acid sequence identity. No group II introns were found in the *N. inconspicua* chloroplast genome, despite their abundance in the mitochondrial genome and a recent report describing their discovery in the chloroplast of pennate diatom *Toxarium undulatum*^39^. However, the *N. inconspicua* chloroplast genome does contain a Tn3 transposon recombinase related to those typically found in bacteria, but also present in the *Semanivis robusta* chloroplast sequence^40^ at 76% amino acid identity.

Like other diatoms, many sequences from the *N. inconspicua* nuclear genome contain targeting signals for intracellular localization to mitochondrial and chloroplast compartments. 1600 mitochondrial target candidates were predicted by MitoFates^41^, and 2172 chloroplast target candidates were predicted by ASAfind^42^. Some of these genes may have been transferred from ancestral organelles and their symbiont predecessors to the nucleus, a process that is believed to be ongoing even in current times^43,44^. However, no evidence of recent intracellular transfer was detected in *N. inconspicua* genes predicted to contain organelle-targeting sequences; the closest GenBank sequence matches for these genes were all to nuclear genomes from other diatoms.

### Shared, unique and expanded protein families

**Figure 6:**
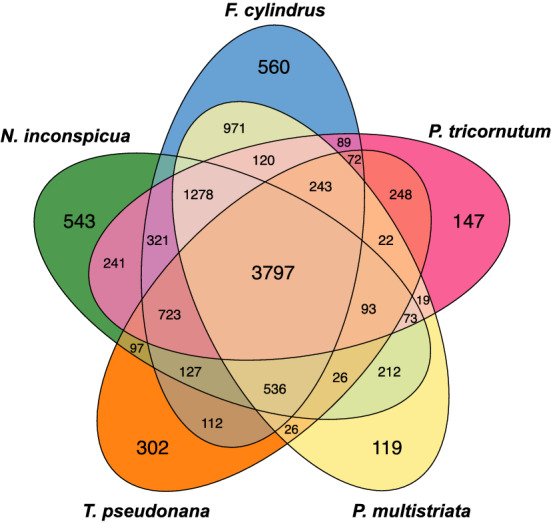
Orthologous protein families in *N. inconspicua* versus other diatom genomes. Allelic duplicates and low-complexity repeat regions were removed from *N. inconspicua* before clustering, leaving 17,968 haploid representatives. Numbers indicate protein families with at least two members, excluding "singletons" that have no orthologous or paralogous matches. Accession numbers associated with each family are provided in Supplementary Dataset 1, obtained from the PhycoCosm haploid gene catalog^86^ for *N. inconspicua,* and complete Genbank genome records for *F. cylindrus* (FMBT00000000.1), *P. tricornutum* (ABQD00000000.1), *P. multistriata* (CAACVS000000000.1), and *T. pseudonana* (AAFD00000000.2).

Predicted protein sequences from *N. inconspicua* were clustered together with those of four other diatom genomes (*F. cylindrus*, *P. multistriata*, *P. tricornutum,* and *T. pseudonana)* to identify shared versus unique families (Fig. 6). 3,797 protein families were shared among all five species, a number similar to the 3,742 families previously reported as shared between three *Pseudo-nitzschia* transcriptomes and the genomes of *P. tricornutum,* and *T. pseudonana*^45^.

Shared families encoded complete enzyme sets for many well-characterized, highly conserved metabolic pathways, including glycolysis, gluconeogenesis, citric acid cycle, C-4 metabolism, oxidative and reductive pentose phosphate pathways, urea cycle, mevalonate and non-mevalonate pathways, and assimilatory sulfate reduction. Complete, paralogous sets of glycolysis and fatty acid pathway enzymes were predicted to be localized in both cytosolic and chloroplast cellular compartments of *N. inconspicua*, along with mitochondrial duplication of the second half of the glycolysis pathway, similar to other diatom genomes where compartmental redundancies have been proposed to enable distributed cellular mechanisms for increasing total metabolic capacity^46–48^.

Functions for 46% of the shared protein families and 67% of those unique to *N. inconspicua* were annotated as hypothetical, while many others were described only by general similarity to PFAM domain patterns. While some cases of apparent divergence within families of indeterminate function might be due to sequencing or assembly errors, transcriptome sequence matches and the existence of closely related allelic copies on syntenic contigs for > 99% of the *N. inconspicua*-unique loci provides strong evidence supporting their validity.

A large number of *N. inconspicua* protein sequences contained domains likely to be involved in the processes of cell-surface adhesion, biofilm formation, and raphe system gliding motility. These domains were identified within the 17,968 haploid gene subset of *N. inconspicua* by protein descriptions containing the keywords fibronectin (n = 171), ankyrin (n = 113), fasciclin (n = 22), laminin (n = 15), outer membrane adhesin (n = 11), lectin (n = 9), von Willebrand factor (n = 3), and villin headpiece domain (n = 2). Several OrthoVenn protein family clusters annotated as fibronectins, outer membrane adhesins, and fasciclins were unique to *N. inconspicua,* without detectable orthologs in the other 4 diatom genomes (Supplementary Data Set 1). Additional conserved diatom loci annotated as capsular and exopolysaccharide biosynthesis proteins, exostosins, and sulfotransferases may participate in the synthesis of sulfated polymer exudates supporting raphid motility. However, no predicted proteins from *N. inconspicua* included the adhesion-specific GDPH pattern recently described in *Semanivis robusta* and the transcriptomes of other benthic diatoms^8^.

*N. inconspicua* tolerates ambient chemistries that are often high in pH and low in dissolved CO_2_, conditions known to limit productivity of aquatic photosynthetic organisms. To understand the molecular basis of this tolerance, we investigated the identities and abundance of several putative components of the biophysical carbon concentrating mechanism (CCM), including bicarbonate transporters and carbonic anhydrases (CAs).

Phylogenetic analysis of bicarbonate transporter sequences from *N. inconspicua* (Supplementary Figure S7) reveals two loci belonging to a metazoan-type clade orthologous to plasma membrane-localized and low CO_2_-sensitive bicarbonate transporters from *P. tricornutum,* as well as a single transporter ortholog predicted to be localized to the *P. tricornutum* chloroplast. Additional bicarbonate transporter family proteins detected using HMM pattern PF00955 belong to a more distant clade, along with other diatom sequences annotated as boron transporters (TC 2.A.31.3,^49^), leading to uncertainty about a potential role in the CCM. Excluding these, *N. inconspicua* has slightly fewer (n = 3 per genome) bicarbonate transporters overall than diatoms on average (n = 4 per genome) suggesting there has been little need to expand bicarbonate acquisition capabilities through genetic diversification in this species.

**Figure 7:**
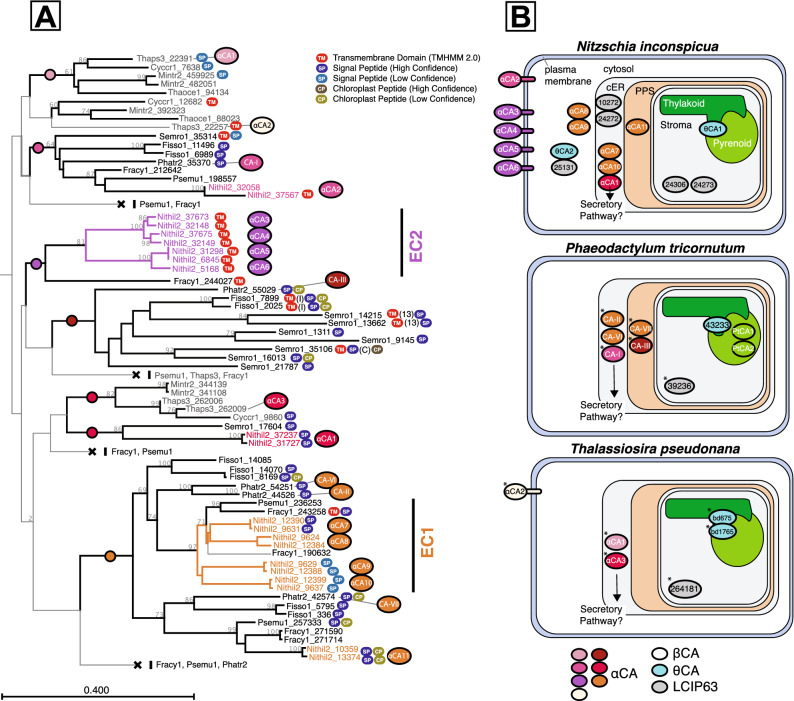
Carbonic anhydrase genes supporting carbon concentration mechanisms. (**A**) Maximum-likelihood phyogeny of putative α-carbonic anhydrases (CAs) from diatom genomes. (**B**) Models of carbon concentrating mechanisms (CCMs) for *N. inconspicua* compared to *P. tricornutum*, and *T. pseudonana* (after^51^), showing predicted subcellular compartment distributions (cER = chloroplast endoplasmic reticulum, PPS = periplastid space). In (**A**), basal branches with higher bootstrap support (> 30) are thicker and denoted with colored circles corresponding to colors in (**B**). Bootstrap values are shown only for nodes with > 60% support. Sequence identifiers are JGI PhycoCosm accessions^86^, with taxonomic abbreviations as defined in Supplementary Fig. 7. Centric diatom species are colored grey, pennate species black, and *N. inconspicua* sequences with the basal branch color, to highlight placement within the diatom phylogeny. Both *N. inconspicua* alleles are shown, when available. Targeting information (Supplementary Dataset 1) is indicated by the following abbreviations: TM, transmembrane domains; SP, signal peptide; CP, chloroplast transit peptide. Asterisks indicate differences from previously proposed CCM models. Chloroplast low confidence predicted genes are assigned to the PPS. Other CA classes (δCA, γCA, ζCA) are not depicted. Protein accession numbers for all species represent entries in the PhycoCosm web portal^86^. Supporting results obtained from SignalP, ASAFind, Mitofates, and TMHMM are provided in Supplementary Dataset 2.

*N. inconspicua* encodes 21 CA genes from the α, γ, δ, θ, and LCIP63 subclasses but does not encode genes from the β, or ζ types (Supplementary Dataset 2,^50^). LCIP63s and αCAs are particularly enriched relative to other diatoms. *N. inconspicua* has six putative LCIP63 candidate loci, three of which are chloroplast-localized, as compared to a single copy each in *P. tricornutum* and *T. pseudonana*^50^, and more than twice the number of αCA loci (n = 11) found in *P. tricornutum* or *T. pseudonana*^51^. Phylogenetic analysis shows this is largely the result of major independent expansions of αCAs belonging to two distinct clades (Fig. 7A). The first expansion (clade EC1) likely occurred prior to the divergence of *N. inconspicua*, *P. multiseries,* and *F. cylindrus,* giving rise to five putatively periplastid space/ER lumen/secretion-targeted genes, while the second expansion, specific to *N. inconspicua* (EC2), led to four paralogous loci each possessing a transmembrane domain in the N-terminus. The only other sequence with an N-terminal transmembrane domain that clades with EC2 is from closely related *F. cylindrus* (Fracy1_244027), making this gene family expansion unique for *N. inconspicua*. A previously described N-terminal transmembrane domain in diatom CAs TpαCA2, TpδCA1, and TpδCA4^52,53^ has been shown experimentally to anchor TpδCA1 in the plasmalemma, supporting the idea that *N. inconspicua* clade EC2 αCA proteins may be similarly targeted to the cell surface^53^.

Predicted identities and subcellular localizations of *N. inconspicua* CCM components were used to construct a cellular model for comparison with existing models of *T. pseudonana* and *P. tricornutum* (Fig. 7B, ^51,54^). The most notable differences between *N. inconspicua* and other diatoms are i) extra copies of putative chloroplast-localized LCIP63 genes, and ii) independent gene family expansions in *N. inconspicua*, giving rise to extra αCA copies that either decorate the cell surface, surround the chloroplast, or are possibly secreted extracellularly. Operating as a CCM, these extra CAs should capture and concentrate CO_2_ towards the pyrenoid, the site of RuBisCO and carbon fixation, in order to sustain high productivity at low concentrations of dissolved CO_2_^55^. Future experimental work is needed to conclusively determine the localization and roles of these CCM components. Nonetheless, these expansions provide good genomic evidence for the presence of a unique and powerful CCM and intracellular pH buffering system relative to what is observed in other diatoms, which is likely to be one of the features underlying tolerance and high productivity over a spectrum of salinities and dissolved CO_2_ levels.

Based on reports of antimicrobial activity in some *Nitzschia* strains^56–58^, bioinformatic searches were performed to identify potential biosynthetic gene clusters that might encode secondary metabolite pathways. AntiSMASH^59^ detected a number of individual genes similar to those commonly associated with biosynthetic gene clusters, including Type III polyketide synthases, non-ribosomal peptide synthetase-like proteins, cytochrome P450s, and isoprenoid synthesis enzymes. However, none of these protein families were identified as unique to *N. inconspicua*, and none of these genes were recognized as belonging to multi-gene clusters similar to those of known secondary metabolites. A separate blast search for genes involved in the production of domoic acid in many strains of *Pseudo-nitzschia*^60^ produced no matches in *N. inconspicua*.

## Discussion

This work describes a new reference-quality, diploid nuclear genome for *Nitzschia inconspicua*. The haploid size of this genome is nearly double that of well-characterized model diatom *P. tricornutum*, but 15–60% smaller than closer pennate relatives *P. multistriata* and *F. cylindrus*. An estimate of 14 chromosome pairs, supported by syntenic alignments of contig pairs with telomer-capped ends, will need to be verified by physical measurements. However, this number is similar to cytological observations of 15–17 chromosomes for other *Nitzschia* species, within the much wider range (8–130) previously reported for more distantly related pennate taxa^61,62^.

The expansion of Class II versus Class I transposable elements in *N. inconspicua* suggests lineage-specific activities promoting enhanced genomic plasticity. This plasticity may be expanded in candidate chromosome pairs 13 and 14, where these elements are particularly abundant. Group II introns like those found in the especially large mitochondrial genome could serve a similar plasticity-enhancing function in this organelle, consistent with widely varying mitochondrial genome sizes reported among other sequenced diatoms^7,36^. The *N. inconspicua* chloroplast genome conforms to the more uniform size characteristic of other diatoms, including a well-conserved overall structure similar to that found in many plants.

Some types of information provided by the *N. inconspicua* diploid genome could not have been obtained from more fragmented, less complete assemblies. Paired coding sequences covering 93% of the genome enabled a direct calculation of allelic heterozygosity at 2.7% (97.3% nucleotide identity), contrasting sharply with the estimate of 25% heterozygosity reported for *F. cylindrus*, although the latter value was calculated from only 5,400 allelic pairs, representing less than 30% of predicted coding sequences^10^. Differences between relatively complete and highly fragmented assemblies may also affect coding sequence percentages, which were calculated at 59% for *N. inconspicua*, 54% for *P. tricornutum*, and 50% for *T. pseudonana*, versus 34% for *P. multistriata and 27%* for *F. cylindrus*. It is not clear if these disparities represent true allelic variability or potential assembly artifacts, but the latter explanation is favored by the lower percentages of complete coding sequences (bounded by canonical start and stop codons) found in diatom genomes with larger numbers of short contigs and lower N50 values (Table 1).

Telomer sequences have long been the subject of intense interest with respect to vertebrate cellular aging, but have not yet been systematically studied in diatoms, and only recently investigated in algae^28^. The unusually G-rich, degenerate pattern of telomer repeats found in *N. inconspicua* was surprising in light of the unremarkable, uniform telomeres found in model diatoms *P. tricornutum* and *T. pseudonana*. The altered nucleotide composition of the *N. inconspicua* repeat pattern should increase secondary structure stability in telomer caps protecting single-stranded chromosome ends, especially at elevated temperatures or under high pH conditions. It will be interesting to learn whether similar degenerate, G-rich motifs occur in other diatoms, and whether they might serve an adaptive function.

*N. inconspicua* genome sequencing has revealed a number of features that may contribute to successful exploitation of benthic habitats and suitability for commercial biofuel production. Like other diatoms, distributed duplication of glycolysis and fatty acid synthesis among different cellular compartments could provide an energy boost enhancing competitive success against other organisms in mixed cultures. *N. inconspicua*'s extensive repertoire of adhesive domain proteins may facilitate attachment to suspended particles during sediment mixing, increasing access to surface light and enhancing photosynthetic efficiency in the shallow, turbid environments where it thrives. Expansion and diversification of carbonic anhydrase paralogs may enable fine-tuning of carbon concentration activities under variable environmental conditions. Resistance to predation and bacterial pathogenesis may be achieved through currently uncharacterized secondary metabolite compounds, produced using genes encoding type III polyketide synthases, ribosomally-produced post-translationally modified proteins, cytochrome P450s, and isoprenoid synthesis enzymes.

The nearly complete set of diploid information delivered by the *N. inconspicua* genome provides a robust new set of reference material for future functional and transcriptomic studies illuminating the physiology of benthic pennate diatoms and harnessing their unique adaptions to support commercial algae biomass and bioproduct production.

## Materials and methods

### Nucleic acid isolation, library construction, and sequencing and assembly

An axenic *N. inconspicua* culture was obtained from a single colony on a streaked agar plate, then scaled up to a 500 mL liquid culture in an artificial brackish growth medium similar to that previously described^63,64^. After 36 h of growth under continuous light, cells were harvested at late exponential phase via centrifugation. High molecular weight DNA was extracted using CTAB-chloroform:isoamyl alcohol^65^. A large-insert (10–20 kb) genomic library for PacBio sequencing was constructed with the SMRTbell Express Template Prep Kit 2.0 (Pacific Biosciences) and sequenced on a single SMRT Cell on the PacBio Sequel platform at the University of California Davis Genome Center DNA Technologies Core.

PacBio sequencing produced 646,344 reads that were cleaned, trimmed, and assembled with CANU v.1.8^18^, using default program parameters except for genomeSize = 175m. This assembly produced contigs ranging in size from 3,477—6,574,884 nucleotides, with an N50 of 3.7 MB. Read mapping to assembled contigs, coverage statistics, and predicted circularity were obtained from CANU program output files. Contigs supported by only a single PacBio read (1X coverage) were discarded.

Nuclear contigs were distinguished from those derived from mitochondria and chloroplasts on the basis of higher G + C nucleotide composition, lower coverage depths, and predicted linearity versus circularity. Length weighted average G + C values for nuclear, chloroplast, and mitochondrial contigs were 45.1%, and 33.4%, and 29.3%, respectively, while coverage depths were 59X, 364X, and 222X. Contig assignments to nuclear, chloroplast, or mitochondrial bins were confirmed by BLASTX searches versus Genbank reference sequences for chloroplast and mitochondrial genomes. Randomly selected, mapped reads from circular organelle contigs were also used for re-assembly at a range of lower coverage depths (12-90X) with more stringent CANU program parameters (correctedErrorRate = 0.01, minOverlapLength = 1000, min_read_length = 20,000).

Transcriptome data to support gene model predictions was obtained using Illumina PE-150 reads from cultures obtained from six *N. inconspicua* cultures grown under conditions of varying light and nutrient availability (Supplementary Table S3). The cells were collected by centrifugation (2,000 × g for 4 min, 4 °C), flash frozen in liquid N_2_ and stored at -80 °C prior to RNA isolation. Total RNA was collected from each sample using TRIzol followed by a Zymo RNA Clean and concentrate kit. Quality was confirmed with an Agilent Bioanalyzer and RNA was treated with DNase to remove contaminating DNA. RNA sequencing was carried out by GENEWIZ (South Plainfield, NJ) using the Illumina HiSeq platform. Raw Illumina reads were cleaned using Trimmomatic version 0.36^66^ with the following program parameters: ILLUMINACLIP:TruSeq3-PE.fa:2:30:10; LEADING:3; TRAILING:10; HEADCROP:15; SLIDINGWINDOW:4:15; MINLEN:120. Cleaned, trimmed transcriptome reads were assembled using Trinity v2.8.4^67^ using default parameters to produce 452,784 contigs.

### Gene models and functional annotation

Organelle genomes were annotated using GeSeq^68^, supported by reference sequences from *P. multiseries* (NC_027265.1), *Halamphora coffeaeformis* (NC_037727.1), *P. tricornutum* (NC_016739.1), and *F. cylindrus* (NC_045244.1). Nuclear genome models were obtained using BRAKER2^21^, with the *F. cylindrus* genome as a seed training model and the Trinity-assembled *N. inconspicua* transcriptome contigs as reference sequences for two subsequent iterations. The number of gene model coding sequences beginning and ending with canonical start (ATG) and termination (TAG, TAA, TGA) codons was tallied using a custom perl script (count_cds_partials.pl, code provided in Supplementary Materials). BLASTN searches were used to measure agreement between predicted coding regions and the original assembled transcriptome sequences used as BRAKER2 input.

Nuclear genome completeness was assessed using BUSCO v.4.06^69,70^ under the taxonomic setting "stramenopiles". Functional gene descriptions were assigned to predicted nuclear proteins based on BLAST matches to reference database sequences at an e-value cutoff of 1e-3 and HMM pattern matches above model-specified gathering cutoffs. Selection of product descriptive names was prioritized in the following order: blast matches to COGs, 2014 update^71^, followed by HMM pattern matches to TIGRFAM v. 15.0^72^ and PFAM v. 32^73^. Candidate transporter proteins were identified based on BLAST searches against the Transporter Classification Database^49^ and PFAM v.32 models bearing the following descriptive terms: transport, permease, efflux, export, antiporter, channel, and porin. KEGG metabolic pathway associations were assigned using KoFAM Koala^74^ and EnrichM v. 0.2.1^75^. Biosynthetic gene cluster searches were performed using AntiSMASH v5^59^ with both plant (plantiSMASH) and bacterial options.

RNA genes were annotated using RNAmmer v. 1.2^76^, Infernal v. 1.1.2^77^ with RFAM.cm database v. 14.1, and tRNAscanSE v 2.07^78^. Subcellular localizations of nuclear proteins were predicted using Hectar^79^, ASAFind^42^, MitoFates^41^, SignalP v. 5.0^80^, SignalP v. 4.1^81^, SignalP v. 3.0^82^, and TMHMM v. 2.0^83^. Numbers of transposon and repetitive DNA sequences were annotated using all curated models from the DFAM database of repetitive DNA families, release 3.2^84^, together with transposase-associated patterns from PFAM v. 32^73^. Total percentages of genomic nucleic acid residues present as low-complexity repeats were determined using RepeatModeler2^24^. Tandemly repeated telomer sequences were identified using BioSerf^85^.

Candidate bicarbonate transporters and carbonic anhydrases (CA) were retrieved using PhycoCosm gene catalogs^49,86^, HMM searches with models PF00955, PF00194, and KOG0382, and BLAST searches against previously annotated CCM components^50–55,87,88^. Full-length sequence alignments and maximum likelihood trees were constructed for bicarbonate transporter and αCA sequences using the CLC Genomics Workbench v. 11.0, set at bootstrap = 100 and protein substitution model = WAG.

### Taxonomic classification

Light micrographs for axenic cultures were taken under bright field DIC and under fluorescence using a Zeiss Imager.A2 (63 × oil objective). Frustules were visualized by acid washing according to^89^. Specimens were prepared for Scanning Electron Microscopy (SEM) as described in^90^. Digital SEM images were acquired with a Hitachi S-4800 Field Emission Scanning Electron Microscope at an accelerating voltage of 5.0 kV.

Previously identified Bacillaceae family nucleic acid sequences for 18S rRNA, 23S rRNA, *psbC*, and *rbcL* genes^17^ were downloaded from Genbank, using the accession numbers provided in Supplementary Table S5. Alignments were first created for each individual gene type using MUSCLE v3.8.31^91^. Gapped fasta format alignment sequences for each individual taxonomic strain were then concatenated and re-aligned with MUSCLE. Multi-locus trees were constructed from these concatenated alignments using FastTree^92^.

### Genome polymorphism, diploidy and allelic variation

Allelic duplicates, alternative transcript predictions, and low complexity sequences were removed from the 38,601 predicted proteins of the whole diploid assembly using the JGI Eukaryotic Annotation Pipeline^86^ to create a set of 17,968 haploid representatives. To identify, compare, and visualize orthologous protein families, these representatives, downloaded from the *N. inconspicua* gene catalog on the PhycoCosm web portal^86^, were clustered together with predicted proteins from haploid genomes of other diatoms downloaded NCBI Genbank using the OrthoVenn2 web server^93^ with an e-value cutoff of 1e-5 and an inflation value of 1.5.

Nuclear genome heterozygosity was estimated based on 17mer counts obtained using Jellyfish version 2.2.10^94^ from both assembled contigs and raw reads, and visualized by the method of Kajitani^95^, as implemented on GenomeScope^96^. Diploid contig pairs were identified by self-BLASTN nucleotide searches and mapping using the NUCMER module of MUMmer version 3.23^97^. Intracellular gene transfer candidates were identified using DarkHorse version 2.0^98,99^ based on taxonomic matches to proteins in the GenBank nr database, downloaded January 2019.

## Supplementary Information

Supplementary files.

Supplementary Dataset 1.

Supplementary Dataset 2.

## Data Availability

Full DNA-sequencing and metadata associated with this study have been deposited in NCBI GenBank under the following accession numbers: BioProject PRJNA675887, Biosample SAMN16729385, SRA study SRP304012, WGS nuclear genome JAGRRH000000000, and organellar genome records MW971520 (chloroplast) and MW971521 (mitochondrion). Haploid representative sequences obtained using the JGI Eukaryotic Annotation Pipeline are available from the JGI Algal Genomics Resource PhycoCosm^86^ at https://mycocosm.jgi.doe.gov/Nithil2/Nithil2.info.html.

## Disclaimer

This report was prepared as an account of work sponsored by an agency of the United States Government. Neither the United States Government nor any agency thereof, nor any of their employees, makes any warranty, express or implied, or assumes any legal liability or responsibility for the accuracy, completeness, or usefulness of any information, apparatus, product, or process disclosed, or represents that its use would not infringe privately owned rights. Reference herein to any specific commercial product, process, or service by trade name, trademark, manufacturer, or otherwise does not necessarily constitute or imply its endorsement, recommendation, or favoring by the United States Government or any agency thereof. The views and opinions of authors expressed herein do not necessarily state or reflect those of the United States Government or any agency thereof.
