# Supplementary material for: Diploid genomic architecture of Nitzschia inconspicua, an elite biomass production diatom
Source: Sci Rep. 2021 Aug 2;11:15592. doi: 10.1038/s41598-021-95106-3 (PMC8329260; doi:10.1038/s41598-021-95106-3)
Supplement: Supplementary file 1 — Supplementary files. [file 41598_2021_95106_MOESM1_ESM.pdf]

Supplementary Information

Diploid genomic architecture of *Nitzschia inconspicua*, an elite biomass production diatom

Aaron Oliver, Sheila Podell, Agnieszka Pinowska, Jesse C. Traller, Sarah R. Smith, Ryan McClure, Alex Beliaev, Pavlo Bohutsky, Eric Hill, Ariel Rabines, Hong Zheng, Lisa Zeigler Allen, Igor V. Grigoriev, Alan Kuo, David Hazlebeck, Eric E. Allen

Contents

Supplementary Table S1 2

Supplementary Table S2 3

Supplementary Table S3 4

Supplementary Table S4 5

Supplementary Table S5 9

Supplementary Figure S1 15

Supplementary Figure S2 16

Supplementary Figure S3 30

Supplementary Figure S4 31

Supplementary Figure S5 32

Supplementary Figure S6 33

Supplementary Figure S7 34

Custom code: count_cds_partials.pl 35

References 36

Supp. Table S1. Assembly properties of syntenic paired contigs in the *N. inconspicua* diploid assembly.

pair number	Ref ctg len	Mate ctg(s)	ref ctg length	ref ctg assembly coverage	mate ctg(s) assembly coverage	average nucleotide identity	MUMmer aln len ref ctg	MUMmer pct aln ref ctg	MUMmer aln len mate ctg(s)	MUMmer pct aln mate ctg(s)
pair 1	tig00000111	tig00000107	3,679,959	60.3	60.2	94%	2,790,740	76%	2,808,708	78%
pair 2	tig00044023	tig00000310	2,278,761	60.1	56.8	93%	1,481,604	65%	1,474,187	77%
pair 3	tig00000176	tig00000241	2,972,054	62.5	56.3	93%	1,998,077	67%	2,026,657	78%
pair 4	tig00000204	tig00000247	3,108,069	62.7	58.5	93%	2,184,523	70%	2,154,154	80%
pair 5	tig00007565	tig00044005	2,584,008	60.2	61.9	93%	1,910,698	74%	2,071,516	66%
pair 6	tig00044003	tig00000121	4,242,913	61.1	61.4	94%	3,030,608	71%	3,007,305	85%
pair 7	tig00043997	tig00000071	5,376,619	67.3	60.7	94%	3,656,905	68%	3,734,145	86%
pair 8	tig00007555	tig00007557	292,286	55.7	47.8	100%	246,033	84%	239,662	99%
pair 9	tig00044033	tig00000034, tig00000040	4,610,928	64.4	60.8, 49.7	94%	3,559,477	77%	3,573,060	87%
pair 10	tig00007564	tig00000069, tig00000068	3,170,983	77.8	63.2, 46.6	94%	1,906,200	60%	1,897,237	85%
pair 11	tig00007560	tig00000376, tig00000375, tig00007561, tig00007567	900,008	62.5	47.8, 47.9, 13.9, 51.2	100%	838,710	93%	904,674	98%
pair 12	tig00007562	tig00000158, tig00044009, tig00000168, tig00000162, tig00007563	1,282,887	104.3	33.0, 6.0, 10.5, 38.7, 39.7	100%	474,708	37%	459,813	99%
pair 13	tig00044028	tig00000004, tig00000074, tig00000008	6,574,884	60.8	62.1, 61.1, 54.7	94%	3,009,567	47%	3,263,325	50%
pair 14	tig00000120	tig00000301, tig00000012	3,900,614	61.1	59.4, 61.3	93%	1,425,792	37%	1,405,244	69%

Supp. Table S2. BUSCO completeness statistics for sequenced diatom genomes. Analyses were based on a Stramenopile model, using amino acid sequences for all predicted proteins from each genome.

**Species**	**type**	**num seqs**	**pct complete**	**single-copy**	**dup-licated**	**frag-mented**	**pct missing**	**Groups searched**
Nitzschia inconspicua str. hildebrandi	protein	38,601	100%	8%	92%	0%	0%	100
Fragilariopsis cylindrus CCMP1102	protein	18,121	95%	95%	0%	4%	1%	100
Pseudo-nitzschia multistriata str. B856	protein	12,055	86%	85%	1%	1%	13%	100
Phaeodactylum tricornutum v2.0	protein	10,198	94%	93%	1%	3%	3%	100
Seminavis robusta D6	protein	37,718	99%	92%	7%	0%	1%	100
Fistulifera solaris JPCC DA0580	protein	20,429	97%	14%	83%	1%	2%	100
Thalassiosira pseudonana v3.0	protein	10,608	94%	93%	1%	3%	3%	100

Supp. Table S3. Culture conditions used to obtain *Nitzschia inconspicua* samples for transcriptome analysis.

Condition Name	Condition Details
Control	Global Algae standard growth media, 5mL of *N. inconspicua* (at ~ O.D. of 1.0) grown in a 50 mL conical tube with closed lid at 24 C with shaking at 130 rpm (tubes were horizontal in the shaker to maximize agitation) in incubator in MCDL, exposed to ~20 of light for 4 hours before RNA samples collected.
Low Nitrate	Same as control but with 0.05 g/L (0.589 mM) of $NaNO_3$
No Nitrate	Same as control but lacking $NaNO_3$
Dark	Same as control but tubes were wrapped in tinfoil
Bioreactor	Continuous cultivations in a custom-built photobioreactor[1] equipped with constant LED-based di-chromatic irradiation system. O.D. was held at 0.08 and the light intensity was 10:10E m^{-2} sec^{-1} at the 630:680 nm linear incident irradiance. PBR was operated under turbidostat mode at pH 9.2, temperature of 25°C, 5.5-L culture volume under 250 rpm agitation, sparging at a 1 L min^{-1} rate with nitrogen
PBR Simulated Pond	Continuous cultivations photobioreactor simulating pond growth

Supp. Table S4. Telomere sequences on paired contig ends. Contig pair number refers to table S1. Sequences at contig 5' ends are shown as reverse complements, to facilitate comparison with 3' ends.

telomere seq id	contig pair num.	telomer position	telo-mere len	telo-mere read cover	assembled telomere sequence
tig00000107_3	1	3 prime	350	13.9	TTAGGGTTAGGGTTGGGTTAGGGTTAGGGTTAGGGGTTAGGGTTGGGGTTGGGGTTAGGGTTGGGTTAGGGTTGG<br>GGTTAGGGTTAGGGTTAGGGTTGGGTTAGGGTTGGGGTTAGGGTTGGGGTTAGGGTTGGGGTTTGGGTTGGGGT<br>TAGGGTTGGGGTTAGGGTTGGGTTAGGGTTGGGGTTAGGGTTGGGGTTAGGGTTAGGGTTGGGGTTAGGGTTGG<br>GGTTAGGGTTAGGGTTGGGGTTAGGGTTAGGGTTAGGGTTGGGGTTAGGGTTAGGGTTAGGGTTGGGGTTAGGG<br>TTGGGGTTGGGGTTAGGGTTGGGGTTAGGGTTGGGTTAGGGTTGGGTTAGGGT
tig00000107_5rc	1	5 prime	363	13.0	AGGGTTAGGGTTGGGGTTAGGGTTAGGGTTAGGGTTAGGGTTAGGGTTAGGGTTAGGGTTAGGGTTGGGGTTAGG<br>GTTGGGTTAGGGTTGGGGTTAGGGTTGGGGTTAGGGTTGGGGTTAGGGTTAGGGTTGGGTTAGGGTTGGGTTAG<br>GGTTGGGTTAGGGTTGGGGTTAGGGTTGGGTTGGGGGTTAGGGTTGGGTTAGGGTTGGGGTTGGGGTTAGGGTT<br>GGGGTTGGGTTAGGGTTGGGGTTAGGGTTGGGGTTGGGGTTAGGGTTGGGGTTAGGGTTGGGGTTAGGTTAGGG<br>TTGGGGTTGGGGTTAGGGTTGGGGTTGGGTTGGGTTAGGGTTGGGGTTAGGGTTAGGGTTGGGGTT
tig00000111_3	1	3 prime	396	15.6	AGGGTTAGGGTTGGGTTAGGGTTGGGTTAGGGTTGGGGTTAGGGTTAGGGTTGGGGTTAGGGTTGGGGTTAGGG<br>TTGGGGTTAGGGTTAGGGTTGGGGTTAGGGTTGGGGTTAGGGTTAGGGTTGGGGTTAGGGTTAGGGTTGGGGTTA<br>GGGTTGGGGTTAGGGTTGGGGTTGGTGTTAGGGTTAGGGTTGGGGTTAGGGTTGGGGTTAGGGTTAGGGTTAGG<br>GTTGGGGTTTGGGTTAGGGTTAGGGTTGGGTTAGGGTTGGGGTTAGGGTTAGGGTTGGGGTTAGGGTTGTTAGGG<br>TTAGGGTTGTTAGGGTTAGGGTTGGGGTTAGGGTTGGGGTTAGGGTTGGGGTTAGGGTTGGGGTTAGGGTTGGG<br>GTTAGGGTTGGGGTTGGGGTTAGG
tig00000310_3	2	3 prime	386	12.2	TGGGTTAGGGTTGGGGTTAGGGTTGGGGTTAGGGTTAGGGTTGGGTTAGGGTTGGGGTTAGGGTTGGGGTTAGG<br>GTTAGGGTTGGGGTTAGGGTTAGGGTTGGGGTTAGGGTTAGGGTTGGGGTTAGGGTTGGGGTTAGGGTTGGGGG<br>TTAGGGTTGGGGTTGGGGTTAGGGTTGGGGTTGGGTTAGGGTTGGGGTTAGGGTTGGGGTTAGGGTTGGGGTTA<br>GGGTTGTGGTTAGGGTTAGGGTTGGGTTAGGGTTGGGTTAGGGTTGGGGGTTAGGGTTGGGGTTAGGGTTGGTTA<br>GGGTTGGGGTTAGGGTTAGGGTTGGGGTTAGGGTTGGGGTTAGGGTTAGGGTTGGGGTTGGGGTTGGGGTTGGG<br>GTTAGGGTTGGGGTT
tig00044023_3	2	3 prime	346	3.5	GGGTTAGGGTTGGGGTTAGGGTTGGGGTTAGGGTTGGGGTTAGGGTTGGGGTTGGGTTGGGGGTTAGGGTTGGG<br>GTTAGGTTGGGGTTAGGGTTGGGGTTAGGGTTGGGTTAGGGTTGGGGTTAGGGTTGGGTTAGGGTTGGGTTAGG<br>GTTGGGGTTGGGGTTGGGGGTTGGGGTTAGGGTTGGGTTAGGGTTGGGGTTAGGGTTGGGGTTAGGGTTGGGGT<br>TAGGGTTGGGGGTTAGGGTTGGGTTAGGGTTGGGTTAGGGTTGGGGTTAGGGTTGGGGTTAGGGTTGGGGTTAG<br>GGTTGGGGTTGGGGTTAGGGTTGGGGTTAGGGTTGGGGTTAGGGTTGGGG
tig00044023_5rc	2	5 prime	385	23.9	TTAGGGTTAGGGTTAGGGTTAGGGTTGGGGTTAGGGTTGAGGGTTAGGGTTAGGGTTGAGGGTTAGGGTTGGGGT<br>TGGGGTTGGGGTTAGGGTTAGGGTTGGGGTTAGGGTTAGGGTTGGGTTAGGGTTGGGGTTAGGGTTGGGTTAGG<br>GTTGGGGTTGGGGTTGGGGTTAGGGTTGGGGTTAGGGTTGGGGTTAGGGTTGGGGTTAGGGTTGGGGTTAGGGT<br>TGGGGTTGGGGTTAGGGTTGGGGTTAGGGTTAGGGTTGGGTTAGGGTTGGGGTTAGGGTTGGGGTTAGGGTTGG<br>GTTGAGGGGTTAGGGTTGGGGTTAGGGTTGGGGTTAGGGTTGGGGTTGGGGTTAGGGTTGGGTTAGGGTTGGGG<br>TTGGGGTTAGGGTT
tig00000176_3	3	3 prime	353	24.2	TTAGGGTTGGGGTTAGGGTTGGGGTTAGGGTTAGGGTTGGGGTTAGGGTTGGGGTTAGGGTTGGGGTTAGGGTT<br>GGGGTTAGGGTTGGGGTTAGGGTTGGGGTTAGGGTTGGGGTTAGGGTTGGGGTTGGGGTTAGGGTTGGGGTTAG<br>GGTTGGGGTTAGGGTTGGGGTTAGGTTGGGGTTAGGGTTGGGGTTAGGGTTGGGTTGGGGTTAGGGTTAGGGTT<br>AGGGTTGGGGGTTAGGGTTGGGGTTGGTGTTAGGGTTGGGTTAGGGTTAGGGTTAGGGTTAGGGTTGGGGTTAG<br>GGTGGGGTTGGGGGTTAGGGTTGGGTTAGGGTTGGGGTTAGGGTTGGGGTTGGGGTT

telomere seq id	contig pair num.	telomer position	telo-mere len	telo-mere read cover	assembled telomere sequence
tig00000176_5rc	3	5 prime	361	5.9	TTAGGGTTGGGGTTAGGGTTAGGGTTGGGTTGGGTTGGGGTTAGGGTTAGGGTTGGGGTTAGGGTTGGGTTAGG<br>GTTGGGGTTAGGGTTGGGGTTGGGGTTAGGGTTGGGTTGGGTTAGGGTTGGGGTTAGGGTTGGGGTTAGGGTTA<br>GGGTTGGGGTTAGGGTTGGGGTTAGGGTTGGGGTTAGGGTTGGGTTAGGGTTGGGTTAGGGTTGGGGTTAGGGT<br>TAGGGTTGGGGTTAGGGTTGGGGTTAGGGTTGGGGTGTTAGGGTTAGGGTTGGGTTAGGGTTGGGGTTAGGGTT<br>GGGGTTAGGGTTGGGTTGGGTTAGGGTTGGGTTAGGTTGGGGTTGGGGTTAGGGTTAGGGTTAGG
tig00000204_3	4	3 prime	346	2.4	TTAGGGTTGGGGTTAGGGTTGGGGTTAGGGTTGGGGTTAGGGTTGGGGTTAGGGTTAGGGTTGGGGTTAGGTTG<br>GGGGTTAGGGTTGGGGTTAGGGTTGGGTTAGGGGTTGGGTTAGGGTCGGGTTTAGGGTTGGGGTTAGGGTTGGG<br>GTTAGGGTTGGGGTTAGGGTTAGGGTTGGGGTTAGGGTTGGGGTTGGGGTTGGGGTTGGGGTTAGGGTTAGGGT<br>TGGGGTTGGGTTGTTAGGGTTGTTAGGGTTAGGGTTGGGGTTGGGGTTGGGGTTGGGGTTAGGGTTGGGGTTAG<br>GGTTGGGGTTAGGGTTGGGGTTTGGGGTTGGGGTTAGGGTTAGGGTTGGG
tig00000204_5rc	4	5 prime	382	27.7	GTTAGGGTTGGGGTTAGGGTTGGGGTTAGGGTTGGGTTAGGGTTGGGGTTAGGGTTGGGTTAGGGTTGGGGTTA<br>GGGTTAGGGTTGGGGTTAGGGTTGGGGTTAGGGTTGGGGTTAGGGTTGGGGTTAGGGTTAGGGTTGGGGTTAGG<br>GTTGGGGTTGGTTAGGGTTGGGGTTAGGGTTAGGGTTGGGTTAGGGTTGGGGTTAGGGTTGGGGTTAGGGTTGG<br>GTTAGGGTTAGGGTTGGGGTTAGGGTTAGGGTTGGGGTTAGGGTTGGGTTAGGGTTGGGTTAGGGTTGGGGTTAG<br>GGTTGTTAGGGTTGGGTTGGGTTAGGGTTGGGGTTAGGGTTGGGGTTAGGGTTAGGGTTAGGGTTGGGTTAGGGT<br>TGGGGTTGGG
tig00000247_5rc	4	5 prime	414	13.9	GGGTTAGGGTTGGGTTAGGGTTGGGGTTAGGGTTGGGGTTAGGGTTGGGGTTAGGGTTGGGTTAGGGTTAGGGT<br>TGGGGTTGGGGTTAGGGTTGGGGTTAGGGTTGGGTTAGGGTTGGGGTTAGGGTTAGGGTTGGGGTTAGGGTTGG<br>GGTTAGGGTTGGGGTTAGGGTTGGGTTAGGGTTAGGTTGGGGTTGGGGTTAGGGTTGGGGTTAGGGTTGGGGTT<br>AGGGTTGGGGTTAGGGTTGGGGTTGGGGTTGGGGTTAGGGTTGAGGGTTAGGGTTGGGTTAGGGTTGGGGTTGG<br>GGTTGGGGTTAGGGTTGGGGTTAGGGTTGGGGTTAGGGTTGGGGTTGGGGTTAGGGTTGGGGTTAGGGTTGGGT<br>TAGGGTTAGGGTTGGGTTAGGGTTGGGGTTGGGTTAGGGTTGGG
tig00007565_5rc	5	5 prime	348	18.5	AATGGGTTAGGGTTGGGGTTGGGGTTGGGGTTGGGGTTAGGGTTTGGGGTTAGGGTTGGGGTTAGGGTTGGGTT<br>AGGGTTGGGGTTAGGGTTGGGGTTAGGGTTAGGGTTAGGGTTAGGGTTAGGGTTGGGGTTGGGGTTAG<br>GGTTGGGGTTAGGGTTGGGGTTGGGGTTGGGGTTAGGGTTGGGGTTAGGGTTGGGGTTAGGGTTAGGGTTGGGG<br>TTGGGTTAGGGTTGGGGTTAGGGTTGGGTTAGGGTTGGGTTAGGGTTAGGTTGGGGTTAGGGTTGGGGTTGGGG<br>TTAGGGTTGGGGTTAGGGTTGGGTTGGGGTTAGGGTTGGGGTTAGGGTTGGG
tig00044005_3	5	3 prime	407	11.7	AGGGTTAGGGTTAGGGTTGGGGTTAGGGTTGGGGTTAGGGTTGGGGTTAGGGTTGGGGTTAGGGTTGGGGTTAG<br>GGTTGGGGTTAGGGTTGGGGTTAGGGTTAGGGTTGGGGTTAGGGTTGGGTTAGGGTTGGGGTTAGGGTTAGGGT<br>TGGGTTAGGGTTGGGGTTAGGGTTAGGGTTGGGGTTGGGGTTGGGGTTTAGGGTTAGGGTTGGGGTTAGGGTTG<br>GGGTTAGGGTTGGGGTTAGGGTTGGGTTAGGGTTGGGTTAGGGTTGGGGTTAGGGTTGGGGTTAGGGTTGGGGG<br>GTTTAGGGTTAGGGTTGGGGTTAGGGTTGGGGGTTAGGGTTGGGGTTGGGGGGTTTAGGGTTGGGGTTAGGGTT<br>GGGGTTGGGTTAGGGTTGGGGTTAGGGTTGGGTTGGG
tig00044005_5rc	5	5 prime	352	12.2	TTAGGGTTGAGGTTAGGGTTGAGGTTAGGGTTGAGGTTAGGGTTGGGGTTAGGGTTGGGTTAGGGTTGGGGTTTG<br>GGTTGGGGTTAGGGTTGGGGTTAGGGTTGGGGTTAGGGTTGGGGTTAGGGTTGGGTTGGGGTTAGGGTTGGGTT<br>AGGTTGGGGTTAGGGTTAGGGTTGGGGTTAGGGTTAGGGTTGGGGTTAGGTTGGGTTAGGGTTAGGGTTGGGGTT<br>AGGGTTGGGGTTAGGGTTGGGGTTAGGGTTGGGGTTGGGGTTAGGGTTGGGGTTAGGGTTGGGTTAGGGTTGGG<br>GTTAGGTTGGGTTAGGGTTAGGGTTGGGGTTAGGGTTGGGTTAGGGTTGGGGTT
tig00000121_3	6	3 prime	358	5.6	AGGTTAGGGTTGGGTTAGGGTTGGGGTTAGGGTTGGGGTTAGGGTTGGGGTTGGGGTTAGGGTTGGGGTTTGGG<br>TTAGGGTTGGGGTTGGGGTTAGGGTTGGGGTTAGGGTTTGGGTTGGGGTTAGGGTTGGGGTTAGGGTTGGGGTT<br>GGGGTTAGGGTTGGGGTTAGGGTTGGGGTTAGGGTTGGGGTTAGGGTTGGGGTTTAGGGTTAGGGTTAGGGTTG<br>GGGTTAGGGTTGGGTTAGGGTTGGGGTTAGGGTTAGGGTTGGGGTTAGGGTTAGGGTTGGGGTTAGGGTTAGGG<br>TTGGGGTTAGGGTTGGGGTTAGGGTTGGGGTTGGGGTTAGGGTTGGGGTTAGGGTTGGGGTT

telomere seq id	contig pair num.	telomer position	telo-mere len	telo-mere read cover	assembled telomere sequence
tig00044003_5rc	9	5 prime	312	1.0	TTGAGGGTTAGGGTTAGGGTTGGGGTTAGGGTTGGGGTTAGGGTTGGGTTAGGGTTAGGGTTAGGGTTGGGGTTA<br>GGGTTAGGGTTGGGGTTAGGGTTGGGGTTAGGGTTGGGGTTAGGGTTGGGGTTAGGGTTGGGTTAGGGTTAGGG<br>TTGGGGTTAGGGTGGGTTAGGGTTAGGGTTGGGGTTAGGGTTGGGGTTAGGGTTGGGGTTAGGGTTAGGGTTGTT<br>AGGGTTGGGGTTAGGGTTGGGGTTAGGGTTGGGGTTAGGGTTGGGGTTGGGGTTAGGGTTGGGGTTAGGGTTGG<br>GGTTGGGTTAGGTT
tig00043997_3	7	3 prime	263	3.9	GGGTTAGGGTTGGGGTTAGGGTTGGGGTTAGGGTTGGGGTTAGGGTTGGGTTAGGGTTGGGGTTAGGGTTGGGT<br>TAGGGTTGGGTTTGGGTTGGGGTTAGGGTTGGGGTTAGGGTTGGGGTTAGGGTTGGGGTTGGGTTAGGGTTAGG<br>GTTGGGGTTAGGTTGGGGTTAGGGTTAGGGTTTGGGGTTAGGGTTGGGGTTGGGGTTGGGGTTAGGGTTGGGGT<br>TAGGGTTAGGGTTGTTAGGGTTGGGGTTGGGTTGGGTTAGG
tig00000071_3	7	3 prime	249	2.6	TTAGGGTTAGGGTTGGGGGTTGGGGTTAGGGTTGGGGTTAGGGTTGGGGTTAGGGTTGGGGTTAGGTTAGGTTG<br>GGGTTAGGGTTTGGGTTAGGGTTTGGGGTTAGGGTTGGGGTTGGGTTGGGGGTTAGGGTTGGGGTTAGGGTTGG<br>GGTTAGGGTTGGGGTTAGGGTTGGGTTAGGGTTGGGGTTAGGGTTGGGGTTAGGGTTGGGGTTAGGGTTGGGGT<br>TAGGGTTGGGGTTAGGGTTGGGTTAGG
tig00007555_5rc	8	5 prime	272	7.0	AGGGTTAGGGTTGGGGTTAGGGTTGGGGTTAGGGTTGGGGTTAGGGTTGGGGTTAGGGTTGGGGTTAGGGTTGG<br>GTTAGGGTTGGGGTTTGGGTTAGGGTTGGGGTTGGGGTTAGGGTTGGGGTTGTTAGGGTTAGGGTTGGGGTTGG<br>GGTTGGGTTAGGGTTGGGGTTAGGGTTGGGGTTTGGGTTAGGGTTGGGTTAGGGTTGGGGTTAGGGTTGGGGTT<br>AGGGTTGGGGTTAGGGTTGGGGTTAGGGTTGGGGTTAGGGTTGGGGTTGG
tig00007562_5rc	12	5 prime	397	31.5	TGGGTTAGGGTTGGGGTTAGGGTTGGGGTTAGGGTTGGGGTTAGGGTTGGGGTTAGGGTTAGGGTTAGGGTTAG<br>GGTTGGGGTTAGGGTTGGGGTTAGGGTTGGGGTTGGGGTTAGGGTTGGGGTTAGGGTTAGGGTTGGGTTGGGGT<br>TAGGGTTGGGGTTAGGGTTAGGGTTGGGGTTAGGGTTGGGGTTGGGGTTAGGGTTGGGGTTAGGGTTGGGGTTA<br>GGGTTGGGGTTGGGGTTAGGGTTGGGGTTAGGGTTAGGGTTGGGGGTTGGGTTGGGGTTAGGGTTGGGGTTAGG<br>GTTGGGTTAGGGTTGGGGTTAGGGTTGGGGTTAGGGTTGGGGTTAGGGTTGGGTTAGGGTTGGGTTAGGGTTGG<br>GGTTAGGGTTGGGGTTAGGGTTGGGTT
tig00044028_3	13	3 prime	345	2.4	AGGGTTAGGGTTAGGGTTGGGGTTAGGGTTGGGGTTAGGGTTGGGGTTAGGGTTGGGTTAGGGTTGGGGTTAGG<br>GTTGGGGTTGGGGTTAGGGTTGGGGTTAGGGTTGGGGTTAGGGTTAGGGTTGGGGTTAGGGTTAGGGTTAGGGT<br>TAGGGTTAGGGTTGGGGTTAGGGTTGGGGTTAGGGTTGGGGTTAGGGTTGGGGTTAGGGTTAGGGTTGGGGTTA<br>GGGTTAGGGTTAGGGTTAGGGTTGGGGTTAGGGTTGGGGTTTGGGTTGGGGTTAGGGTTGGGGTTGGGGTTAGG<br>GTTAGGGTTGGGGTTAGGGTTGGGTTAGGGTTGGGGTTAGGGTTGGGTT
tig00000004_3	13	3 prime	410	16.8	TTAGGGTTGGGGTTAGGGTTGGGTTAGGGTTGGGGTTAGGGTTGGGTTAGGGTTGGGTTAGGGTTGGGGTTAGG<br>GTTGGGGTTAGGGTTGGGGTTAGGGTTGGGGTTAGGGTTGGGGTTAGGGTTAGGGTTGGGGTTAGGGTTGGGGT<br>TAGGGTTGGGGTTAGGGTTGGGGTTGGGGTTAGGGTTAGGGTTGGGGTTAGGGTTGGGGTTAGGGTTGGGGTTA<br>GGGTTGGGTTAGGGTTGGGGTTAGGGTTAGGGTTGGGGTTGGGGTTAGGGTTGGGGTTAGGGTTGGGGTTGGGG<br>TTAGGGTTGGGTTAGGGTTGTTAGGGTTGGGGTTAGGGTTGGGGTTGGGTTAGGGTTAGGGTTGGGGTTAGGGTT<br>GGGGTTTGGGTTAGGGGGTTAGGGTTGGGGTTAGGGTTG
tig00000074_3	13	3 prime	404	2.8	TAGGGTTAGGGTTGGGGTTAGGGTTAGGGTTGGGGTTAGGGTTGGGGTTAGGGTTAGGGTTAGGGTTGGGGTTA<br>GGGTTGGGGTTAGGGTTGGGGTTAGGGTTGGGGTTAGGGTTAGGGTTGGGGTTAGGGTTAGGGTTGGGGTTGGG<br>GTTAGGGTTTGTTAGGGTTAGGGGGGTTAGGGTTGGGGGTTAGGGTTGGGGTTAGGGTTAGGGTTGGGGTTAGG<br>GTTAGGGTTGGGGTTAGGGTTGGGGTTGGGGTTAGGGTTGGGGTTAGGGTTGGGGTTAGGGTTGGGGTTAGGGT<br>TGGGTTAGGGTTGGGGTTGGGGTTAGGGTTGGGGTTAGGGTTGGGGTTAGGGTTGGGTTAGGGTTGGGGTTTAG<br>GGTTGGGGTTAGGGTTGGGGTTGGGGTTAGGGTT
tig00000074_5rc	13	5 prime	390	14.3	AGGGTTAGGGTTAGGGTTGGGGTTAGGGTTGGGGTTAGGGTTAGGGTTGGGTTAGGGTTGGGTTAGGGTTATTAG<br>GGTTGGTAGGGTTGGGTTTGGGTTGGGGTTAGGGTTGGGGTTAGGGTTGGGTTAGGGTTAGGTTAGGGTTAGGGT<br>TGGGGTTAGGGTTGGGGTTAGGGTTGGGGTTAGGGTTGGGTTAGGGTTGGGGTTAGGGTTGGGGTTAGGGTTGG<br>GTTAGGGTTGGGGTTGGGGTTAGGGTTGGGGTTGGGGTTAGGGTTGGGGTTAGGGTTGGGTTAGGGTTGGGGTT<br>AGGGTTAGGGTTGGGGTTAGGGTTGGGGTTAGGGTTGGGGTTAGGGTTGGGGTTTAGGGTTGGGGTTAGGGTTT<br>GGGGTTAGGGTTGGGGTT

telomere seq id	contig pair num.	telomer position	telo-mere len	telo-mere read cover	assembled telomere sequence
tig00000120_3	14	3 prime	399	23.9	AGGGTTAGGGTTGGGGTTAGGGTTAGGGTTGGGTTAGGGTTAGGGTTAGGGTTGGGGTTAGGGTTAGGGTTGGG<br>GTTAGGGTTGGGGTTGGGGTTGGGGTTAGGGTTGGGGTTAGGGTTGGGGTTGGGGTTGGGGTTGGGGTTGGGG<br>GTTGGGGTTGGGGTTGGGGTTGGGGTTGGGGTTAGGGTTGGGGTTTGGGTTAGGGTTAGGGTTGGGGTTAGGGT<br>TGGGGTTTGGGTTAGGGTTAGGGTTGGGTTGGGGTTAGGGTTGGGGTTAGGGTTGGGTTAGGGTTGGGGTTAGG<br>GTTGGGGTTGGGTTAGGGTTGAGGGTTAGGGTTGGGGTTAGGGTTGGGGTTAGGGTTGGGGTTAGGGTTGGGGT<br>TGGGGTTAGGGTTGGGTTAGGGTTGGGGTT
tig00000301_3	14	3 prime	379	3.2	TAGGGTTAGGTTAGGGTTAGGGTTAGGGTTGGGGTTGGGGTTGAGGGTTAGGGTTAGGGTTGGGGTTGGGGTTA<br>GGGTTGGGGTTAGGGTTGGGGTTAGGGTTAGGGTTGGGGTTGGGGTTAGGGTTAGGGTTGGGGTTAGGGTTGGG<br>TTAGGGTTAGGTTAGGGTTGGGGTTTAGGGTTGGGGTTGGGGTTAGGGTTGGGGTTAGGGTTGGGGTTTAGGGTT<br>AGGGTTAGGGTTGGGTTAGGGTTGGGGTTGGGGTTAGGGTTGGGGTTAGGGTTAGGGTTGGGGTTAGGGTGGGT<br>TAGGGTTGGTGTTAGGGTTGGGGTTAGGGTTGGGGTTAGGGTTGGGTTGGGGTTGGGGTTAGGGTTGGGGGTTA<br>GGGTTGGG
tig00000012_3	14	3 prime	338	3.2	TTAGGGTTAGGGTTGGGGTTAGGGTTGGGGTTAGGGTTAGGGTTAGGTTGGGGTTAGGGTTGGGGTTAGGGTTGG<br>GGTTAGGGTTAGGGTTAGGGTTAGGGTTGGGGTTAGGGTTGGGGTTAGGGTTGGGTTAGGGTTGGGGTTAGGGTT<br>AGGGTTGGGGTTTAGGGTTAGGGTTGGGGTTAGGGTTGGGGTTGGGGTTAGGGTTGGGGTTAGGGTTGGGGTTA<br>GGGTTGGGGTTTAGGGTTGGGGTTAGGGTTGGGGTTAGGGTTGGGGTTAGGGTTGGGGTTAGGGTTGGGTTAGG<br>GTTGGGTTAGGGTTGGGTTGGGGTTAGGGTTGGGGTTGGG

Supp. Table S5. Supp. Table S5. Accession numbers for Bacillariaceae species used in 4-gene multi-locus phylogenetic tree (Supplementary Figure S1)..

Species_name	pbsC id	rbcL id	LSU id	SSU id
Achnanthes_coarctata_UTEX_FD185	HQ912287.1	HQ912458.1	-	HQ912594.1
Achnanthes_sp_MPA-2013_ECT3684Achnan	KC309620.1	KC309548.1	-	KC309476.1
Achnanthes_sp_MPA-2013_ECT3883Achnan	KC309618.1	KC309546.1	-	KC309474.1
Achnanthes_sp_MPA-2013_ECT3911Achnan	KC309619.1	KC309547.1	-	KC309475.1
Achnanthes_sp_MPA-2013_SanNic1Achnan	KC309617.1	KC309545.1	-	KC309473.1
Achnanthes_sp_SZCZCH113	KT943626.1	KT943615.1	-	KT943601.1
Achnanthes_sp_SZCZM119	KT943627.1	KT943616.1	-	KT943602.1
Amphora_laevissima_7314-AMPH085	KJ463494.1	KJ463464.1	-	KJ463434.1
Amphora_obtusa_var_crassa_6951-AMPH070	KJ463496.1	KJ463466.1	-	KJ463436.1
Amphora_vixvisibilis_SZCZCH967	KT943706.1	KT943670.1	-	KT943648.1
Bacillaria_paxillifera_UTEX_FD468	HQ912320.1	HQ912491.1	AF417678.1	HQ912627.1
Bacillaria_sp_BAC901CAT	MN734007.1	MN734075.1	MN750428.1	MN750450.1
Bacillaria_sp_SZCZP613	MN920690.1	MN920681.1	MN944012.1	MN944000.1
Craspedostauros_alyoubyi_UTKSA0083	KX981791.1	KX981814.1	-	KX981857.1
Craspedostauros_cf_neoconstrictus_CCMP1120	KX981793.1	KX981817.1	-	KX981860.1
Craspedostauros_paradoxa_GU44BK-1_keeledHcpA25	KX981792.1	KX981816.1	-	KX981858.1
Cylindrotheca_closterium_CCMP1855	HQ912338.1	HQ912509.1	-	HQ912645.1
Cylindrotheca_sp_SZCZCH50	MN920684.1	MN920674.1	MN944004.1	MN943995.1
Cylindrotheca_sp_SZCZCH690	MN920685.1	MN920676.1	MN944005.1	MN943996.1
Denticula_kuetzingii_UTEX_FD135	HQ912303.1	HQ912474.1	-	HQ912610.1
Diploneis_sp_Coz1_peanut_penn1	KX981795.1	KX981819.1	-	KX981839.1
Eunotia_bilunaris_UTEX_FD412	HQ912292.1	HQ912463.1	-	HQ912599.1
Eunotia_glacialis_UTEX_FD46	HQ912279.1	HQ912450.1	-	HQ912586.1
Eunotia_pectinalis_NIES461	HQ912329.1	HQ912500.1	-	HQ912636.1
Eunotia_sp_MPA-2013_ECT3676Eunotia	KC309623.1	KC309552.1	-	KC309480.1
Fragilariopsis_kerguelensis_E13B2	EF520305.1	EF423500.1	-	-

Species_name	pbsC id	rbcL id	LSU id	SSU id
Fragilariopsis_kerguelensis_L26-C5	EF520306.1	-	-	KJ866919.1
Fragilariopsis_kerguelensis_MM_E13B2	EF520305.1	-	KC832999.1	-
Hantzschia_amphioxys_var_major_A4	HQ912376.1	HQ912390.1	-	HQ912404.1
Nitzschia_acicularis_Nit56	MN734045.1	MN734084.1	KX889110.1	MN750489.1
Nitzschia_acicularis_R20	MN734050.1	KX889095.1	MN750438.1	MN750494.1
Nitzschia_acidoclinata_TCC537	-	KC736602.1	MN696740.1	KC736632.1
Nitzschia_acidoclinata_TCC538	-	MN696763.1	MN696741.1	KT072971.1
Nitzschia_acidoclinata_TCC560	-	MN696765.1	MN696743.1	MN696711.1
Nitzschia_acidoclinata_TCC619	-	MN696770.1	MN696749.1	MN696720.1
Nitzschia_amphibia_RT5	MN734051.1	-	AM182194.1	-
Nitzschia_aurariae_SZCZCH966	KT943698.1	KT943663.1	-	KT943639.1
Nitzschia_capitellata_capitellata_Scot1	MN734010.1	FN557030.1	HF679148.1	MN750453.1
Nitzschia_capitellata_capitellata_Scot2	MN734011.1	FN557031.1	HF679149.1	MN750454.1
Nitzschia_capitellata_capitellata_Spain	MN734012.1	FN557032.1	-	MN750455.1
Nitzschia_capitellata_strain_262	-	-	AM909631.1	-
Nitzschia_cf_aequorea_DM1004CAT	-	HF675062.1	-	-
Nitzschia_cf_aequorea_Nit1004CAT	MN734034.1	-	HF679146.1	MN750480.1
Nitzschia_cf_ardua_L44	MN734030.1	HF675061.1	HF679147.1	MN750476.1
Nitzschia_cf_bulnheimiana_AG	-	HF675063.1	AM183586.1	MN750449.1
Nitzschia_cf_dissipata_var_media_BC0470	MN718802.1	MN718767.1	MN725794.1	MN750422.1
Nitzschia_cf_gracilis_Nit51	MN734043.1	MN734082.1	-	MN750487.1
Nitzschia_cf_microcephala_L56	MN734031.1	HF675103.1	HF679186.1	-
Nitzschia_cf_pusilla_CCMP558	MN734013.1	HF675129.1	HF679204.1	MN750456.1
Nitzschia_cf_pusilla_L1	MN734027.1	-	-	MN750473.1
Nitzschia_cf_pusilla_L25	MN734028.1	-	-	MN750474.1
Nitzschia_cf_pusilla_L3	MN734029.1	-	-	MN750475.1
Nitzschia_cf_pusilla_Nit_44	MN734041.1	HF675119.1	HF679199.1	MN750485.1
Nitzschia_cf_pusilla_NIT1003CAT	MN734033.1	MN734077.1	MN750429.1	MN750479.1
Nitzschia_cf_pusilla_TCC586	-	KT072926.1	MN696747.1	MN696717.1
Nitzschia_cf_pusilla_TCC665	-	MN696772.1	MN696751.1	MN696723.1

Species_name	pbsC id	rbcL id	LSU id	SSU id
Nitzschia_cf_recta_BC0795	MN718804.1	MN718791.1	MN725811.1	MN750424.1
Nitzschia_cf_sigma_NIT1013ABR	MN734038.1	MN734080.1	KX889109.1	MN750484.1
Nitzschia_cf_volvendirostrata_SZCZCH845	KT943700.1	KT943665.1	-	KT943641.1
Nitzschia_costei_TCC521	-	KC736604.1	MN696738.1	-
Nitzschia_costei_TCC550	-	MN696764.1	MN696742.1	MN696710.1
Nitzschia_dissipata_TCC632	KT943700.1	MN696771.1	MN696750.1	MN696722.1
Nitzschia_dissipata_TCC707	-	MN696773.1	MN696753.1	MN696724.1
Nitzschia_draveillensis_Nit50	MN734042.1	MN734081.1	MN750432.1	MN750486.1
Nitzschia_draveillensis_TCC700	-	KC736605.1	MN696752.1	KC736635.1
Nitzschia_dubiiformis_s0311	-	AB430696.1	AB430656.1	AB430616.1
Nitzschia_filiformis_UTEX_FD267	HQ912282.1	HQ912453.1	-	HQ912589.1
Nitzschia_fonticola_C	MN734009.1	-	-	MN750452.1
Nitzschia_fonticola_C-RT26	-	HF675068.1	AM182193.1	-
Nitzschia_fonticola_TCC533	-	KT072921.1	MN696739.1	MN696709.1
Nitzschia_gracilis_TCC576	-	MN696768.1	MN696746.1	MN696715.1
Nitzschia_inconspicua_G1_2	MN734014.1	HF675072.1	HF679152.1	MN750457.1
Nitzschia_inconspicua_G1_3	MN734015.1	HF675073.1	HF679153.1	MN750458.1
Nitzschia_inconspicua_G2_1	MN734016.1	HF675074.1	HF679154.1	MN750459.1
Nitzschia_inconspicua_G2_2	MN734017.1	HF675075.1	HF679155.1	MN750460.1
Nitzschia_inconspicua_G3_1	MN734018.1	HF675080.1	-	MN750461.1
Nitzschia_inconspicua_G3_2	-	HF675081.1	HF679160.1	MN750462.1
Nitzschia_inconspicua_G3_3	MN734019.1	HF675082.1	HF679161.1	MN750463.1
Nitzschia_inconspicua_G3_4	MN734020.1	HF675083.1	-	MN750463.1
Nitzschia_inconspicua_G4_1	MN734021.1	HF675084.1	HF679163.1	MN750465.1
Nitzschia_inconspicua_G4_2	-	HF675085.1	HF679164.1	MN750466.1
Nitzschia_inconspicua_G5_1	MN734022.1	HF675087.1	HF679166.1	MN750467.1
Nitzschia_inconspicua_G5_2	MN734023.1	HF675088.1	HF679167.1	MN750468.1
Nitzschia_inconspicua_G6_1	MN734024.1	HF675102.1	HF679184.1	MN750469.1
Nitzschia_inconspicua_G7_1	-	MN734076.1	HF679185.1	MN750470.1
Nitzschia_inconspicua_TCC474	-	MN696758.1	MN696733.1	MN696705.1

Species_name	pbsC id	rbcL id	LSU id	SSU id
Nitzschia_inconspicua_TCC487	-	KC736607.1	MN696734.1	KC736636.1
Nitzschia_inconspicua_TCC488	-	MN696760.1	MN696735.1	MN696706.1
Nitzschia_inconspicua_TCC498	-	MN696761.1	MN696736.1	MN696707.1
Nitzschia_inconspicua_TCC510	-	MN696762.1	MN696737.1	KT072967.1
Nitzschia_inconspicua_TCC571	-	MN696766.1	MN696744.1	MN696713.1
Nitzschia_lembiformis_R1	-	-	HE798209.1	-
Nitzschia_lembiformis_R2	MN734049.1	KX889094.1	MN750437.1	MN750493.1
Nitzschia_linearis_Nit53	MN734044.1	MN734083.1	MN750433.1	MN750488.1
Nitzschia_microcephala_L56	MN734031.1	-	-	MN750477.1
Nitzschia_microcephala_R10	MN734047.1	MN734086.1	MN750436.1	MN750491.1
Nitzschia_palea_Aitor5	-	-	AM183242.1	-
Nitzschia_palea_BB2b	-	KJ542517.1	AM183240.1	-
Nitzschia_palea_Belgium-1	MN734008.1	-	-	MN750451.1
Nitzschia_palea_Japan_D	MN734025.1	HF675125.1	-	MN750471.1
Nitzschia_palea_Japan_F	MN734026.1	HF675123.1	-	MN750472.1
Nitzschia_palea_Mayama	-	-	AM183233.1	-
Nitzschia_palea_New_Spain2	MN734032.1	HF675127.1	HF679201.1	MN750478.1
Nitzschia_palea_Nit_B2	-	-	AM183245.1	-
Nitzschia_palea_Nit_B4	-	-	AM183247.1	-
Nitzschia_palea_Nit_C	-	-	AM183246.1	-
Nitzschia_palea_R12	MN734048.1	KJ542502.1	KJ542429.1	MN750492.1
Nitzschia_palea_Spain_A2	-	HF675124.1	-	MN750499.1
Nitzschia_palea_Spain_A3	MN734055.1	FN557022.1	-	MN750500.1
Nitzschia_palea_Spain_A4	MN734056.1	FN557023.1	-	MN750501.1
Nitzschia_palea_Spain_C	MN734057.1	FN557025.1	-	MN750502.1
Nitzschia_palea_SriLanka1	-	KJ542515.1	AM183235.1	MN750503.1
Nitzschia_palea_SriLanka2	MN734058.1	KJ542516.1	AM183236.1	MN750504.1
Nitzschia_palea_TCC435	-	KC736609.1	KJ542414.1	KC736638.1
Nitzschia_palea_TCC570	-	KC736610.1	KJ542397.1	KC736639.1
Nitzschia_palea_UK	MN734066.1	-	-	MN750512.1

Species_name	pbsC id	rbcL id	LSU id	SSU id
Nitzschia_palea_Victor_02-9E	MN920688.1	-	AM183248.1	-
Nitzschia_pusilla_L1	-	HF675108.1	HF679193.1	-
Nitzschia_pusilla_L25	-	HF675110.1	HF679196.1	-
Nitzschia_pusilla_L3	-	HF675109.1	HF679195.1	-
Nitzschia_rectilonga_SZCZE431	MN920688.1	MN920679.1	MN944010.1	MN943999.1
Nitzschia_sigmoidea_BC0787	MN718803.1	MN718790.1	MN725810.1	MN750423.1
Nitzschia_soratensis_DM1008MK	-	HF675112.1	HF679197.1	-
Nitzschia_soratensis_DM1009MK	-	HF675111.1	HF679198.1	-
Nitzschia_soratensis_NIT1008KEL	MN734035.1	-	-	MN750481.1
Nitzschia_soratensis_NIT1009KEL	MN734036.1	-	-	MN750482.1
Nitzschia_sp_KSA0035	KU179143.1	KU179116.1	-	KU179128.1
Nitzschia_sp_NIT1012CAT	MN734037.1	MN734079.1	MN750431.1	MN750483.1
Nitzschia_sp_s0819	MN734052.1	HF675116.1	-	MN750496.1
Nitzschia_sp_SZCZCH1090	MN920683.1	MN920673.1	MN944003.1	MN943994.1
Nitzschia_sp_SZCZM117	KU179142.1	KU179115.1	-	KU179129.1
Nitzschia_sp_SZCZP71	MN920691.1	MN920682.1	MN944013.1	MN944001.1
Nitzschia_supralitorea_TCC606	-	MN696769.1	MN696748.1	MN696719.1
Nitzschia_traheaformis_SZCZCH970	KT943701.1	KT943666.1	-	KT943642.1
Nitzschia_traheaformis_SZCZCH971	KT943702.1	KT943667.1	MN944007.1	KT943643.1
Nitzschia_traheaformis_SZCZCH972	MN920686.1	KT943668.1	-	KT943644.1
Nitzschia_tubicola_TCC575	-	MN696767.1	MN696745.1	MN696714.1
Nitzschia_valdestriata_SZCZCH969	KT943699.1	KT943664.1	-	KT943640.1
Nitzschia_varelae_NIT952CAT	MN734046.1	KX889093.1	KX889112.1	MN750490.1
Pleurosigma_sp_UTKSA0019	KX981798.1	KX981822.1	-	KX981840.1
Psammodictyon_constrictum_s0309	-	AB430697.1	AB430657.1	AB430617.1
Psammodictyon_sp_SZCZE328	MN920687.1	MN920677.1	MN944008.1	MN943997.1
Psammodictyon_sp_SZCZE331	-	MN920678.1	MN944009.1	MN943998.1
Psammodictyon_sp_SZCZE459	MN920689.1	MN920680.1	MN944011.1	-
Pseudo-nitzschia_americana_FBJUN06.1.6	EF520312.1	-	EF522108.1	-
Pseudo-nitzschia_americana_FBJun06.6	EF520321.1	EF423504.1	-	-

Species_name	pbsC id	rbcL id	LSU id	SSU id
Pseudo-nitzschia_americana_UNC1412	-	-	-	KX229689.1
Pseudo-nitzschia_delicatissima_CLA1.A1	EF520331.1	EF520340.1	EF522114.1	-
Pseudo-nitzschia_galaxiae_FB06	EF520326.1	EF423509.1	EF522113.1	-
Pseudo-nitzschia_galaxiae_SZN-B606	-	-	-	KJ608078.1
Pseudo-nitzschia_sp_CLA1.D2	EF520318.1	EF520338.1	EF522115.1	-
Staurotropis_americana_Coz4_cfUnd-5	KX981807.1	-	-	KX981854.1
Staurotropis_americana_FishPassMangrove_staurosA1	KX981808.1	KX981834.1	-	KX981855.1
Staurotropis_khiyamii_UTKSA0047	KX981806.1	KX981832.1	-	KX981853.1
Tryblionella_apiculata_TRY946CAT	MN734061.1	MN734089.1	MN750443.1	MN750507.1
Tryblionella_apiculata_TRY947CAT	MN734062.1	MN734090.1	MN750444.1	MN750508.1
Tryblionella_apiculata_UTEX_FD465	HQ912293.1	HQ912464.1	-	HQ912600.1
Tryblionella_cf_compressa_TRY1006CAT	MN734059.1	-	MN750441.1	MN750505.1
Tryblionella_cf_compressa_TRY1007CAT	MN734060.1	MN734088.1	MN750442.1	MN750506.1
Tryblionella_gaoana_SZCZCH97	KT943697.1	KT943683.1	MN944006.1	KT943638.1
Tryblionella_hungarica_TRY951CAT	MN734063.1	MN734091.1	MN750446.1	MN750509.1
Tryblionella_hungarica_TRY981CAT	MN734064.1	MN734092.1	MN750447.1	MN750510.1
Tryblionella_hungarica_TRY986CAT	MN734065.1	MN734093.1	MN750448.1	MN750511.1
Tryblionella_sp_s0863	MN734054.1	HF675117.1	MN750440.1	MN750498.1

Supplementary Figure S1. Placement of *N. inconspicua* in a four-gene (SSU, LSU, rbcL and psbC) phylogenetic tree of the Bacillariaceae family. Accession numbers of sequences used for each organism are presented in Supp. Table S5.

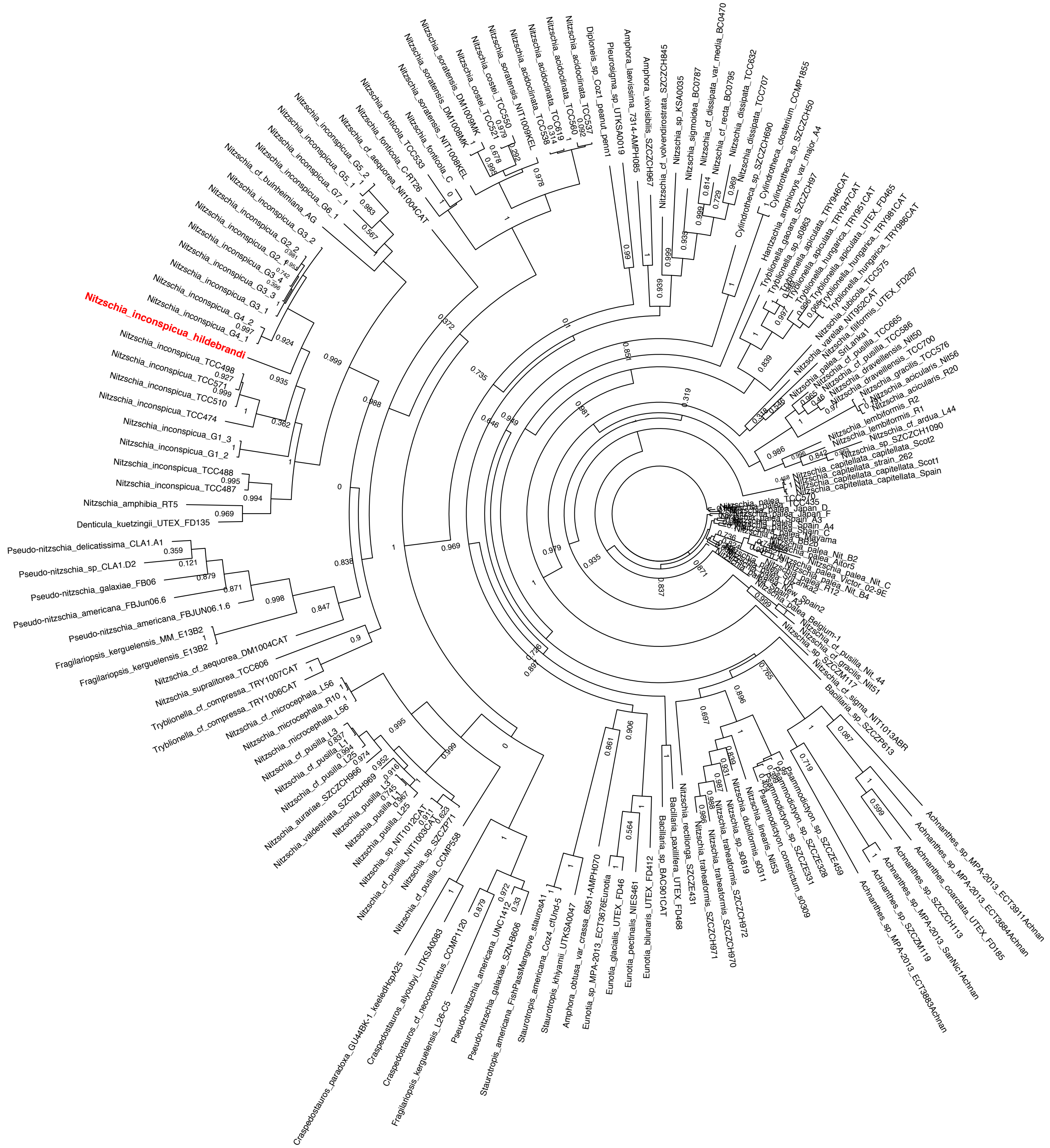

Supplementary Figure 2a. MUMmer dot plot of contig pair #1

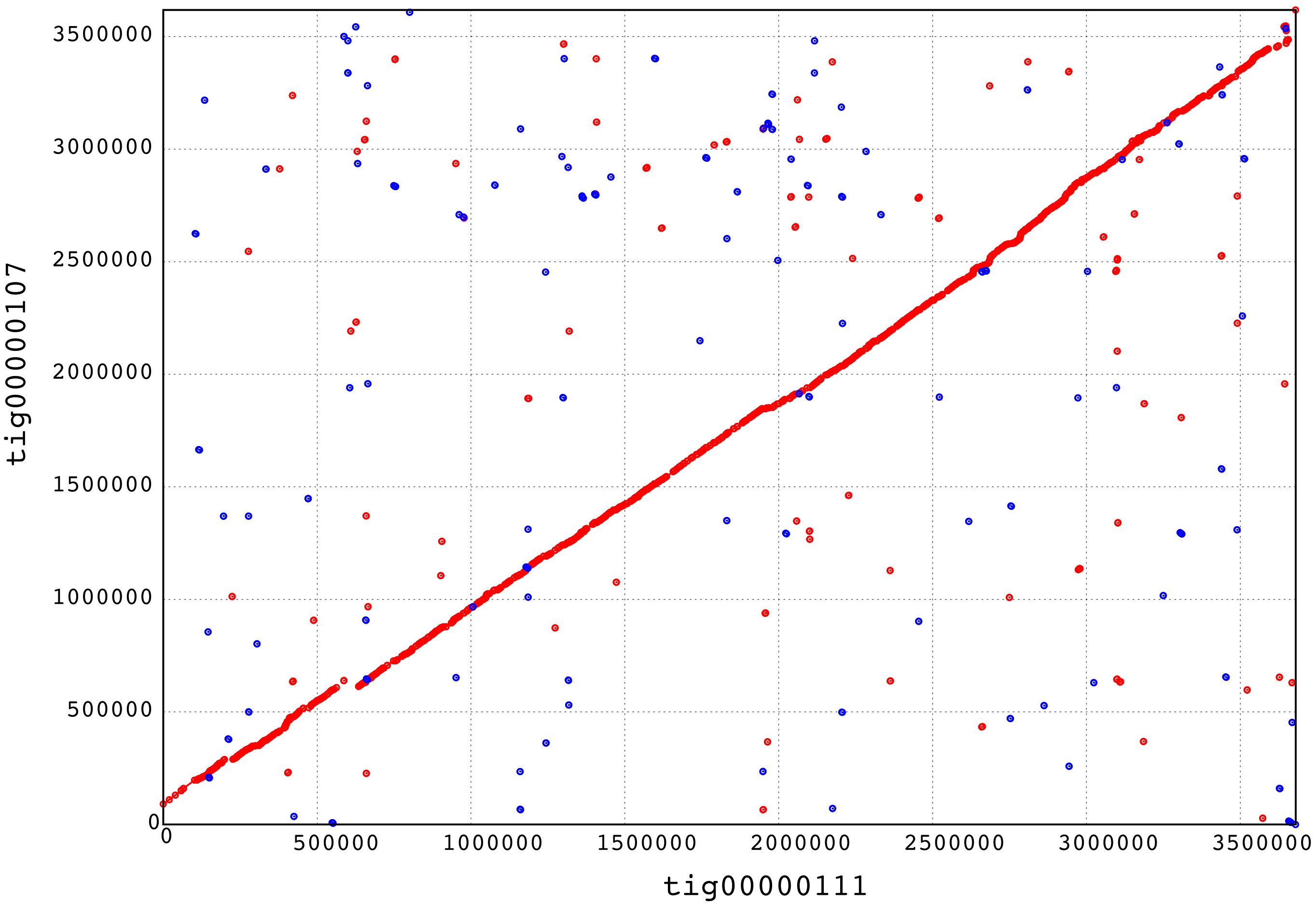

Supplementary Figure 2b. MUMmer dot plot of contig pair #2

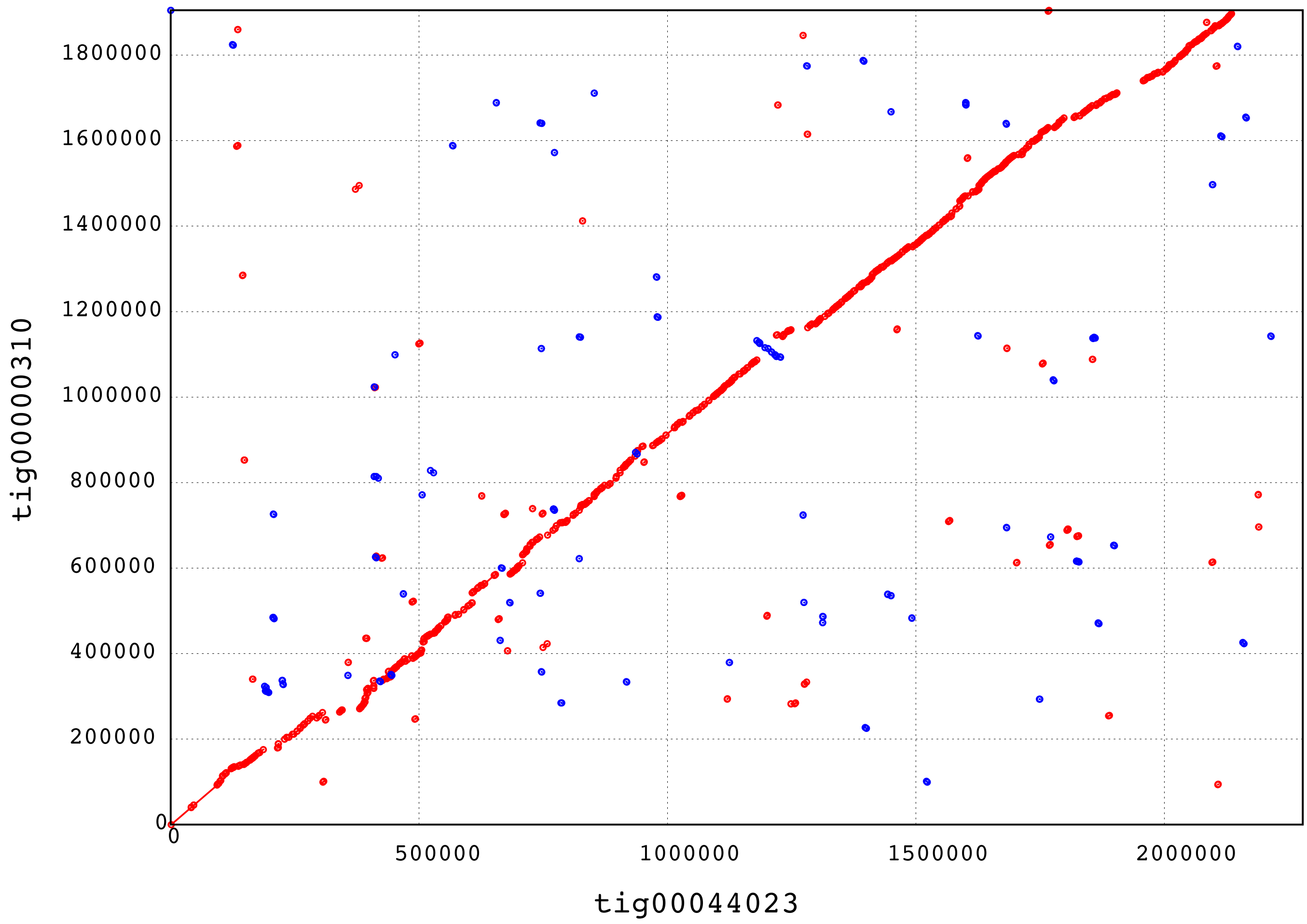

Supplementary Figure 2c. MUMmer dot plot of contig pair #3

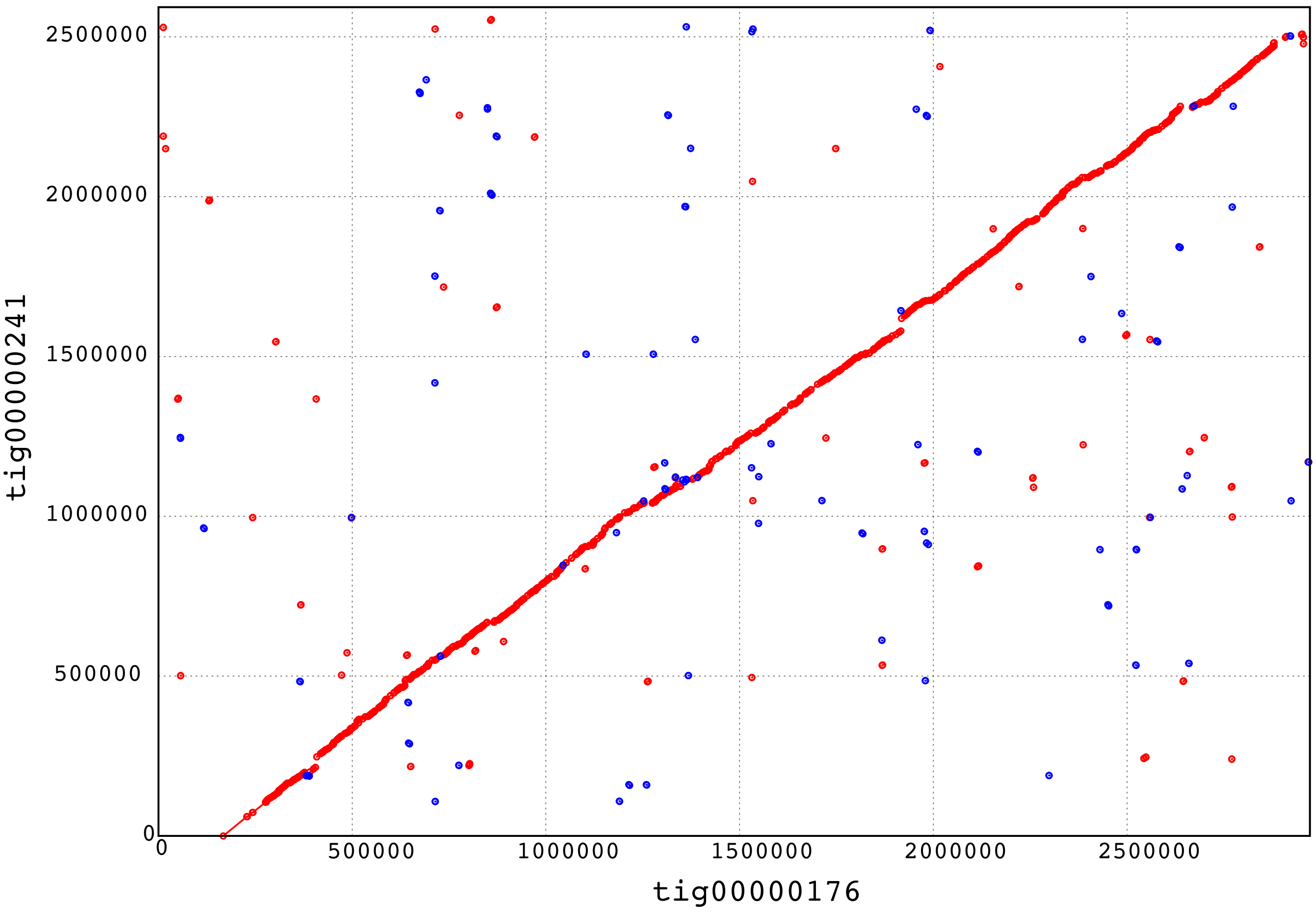

Supplementary Figure 2d. MUMmer dot plot of contig pair #4

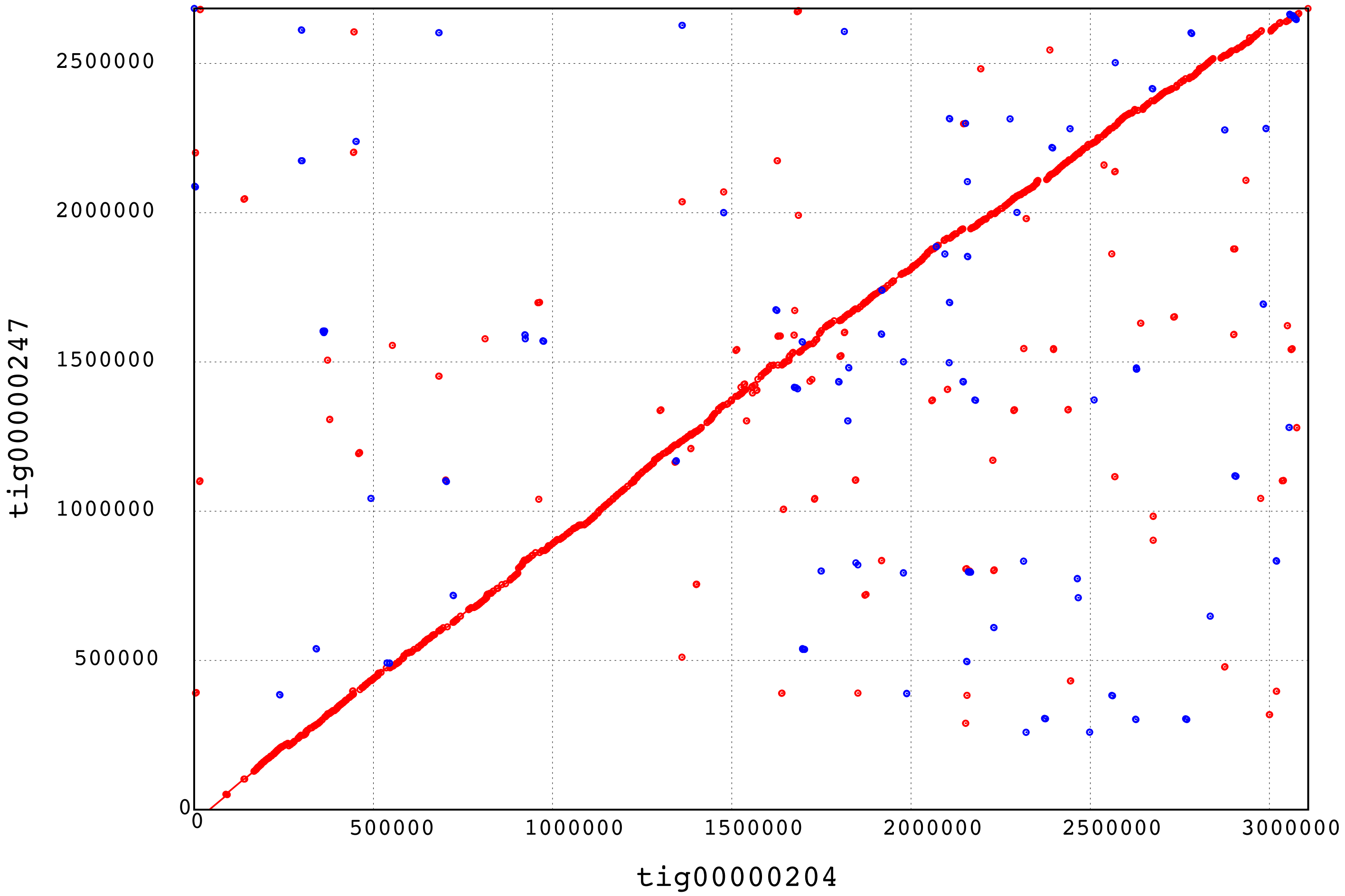

Supplementary Figure 2e. MUMmer dot plot of contig pair #5

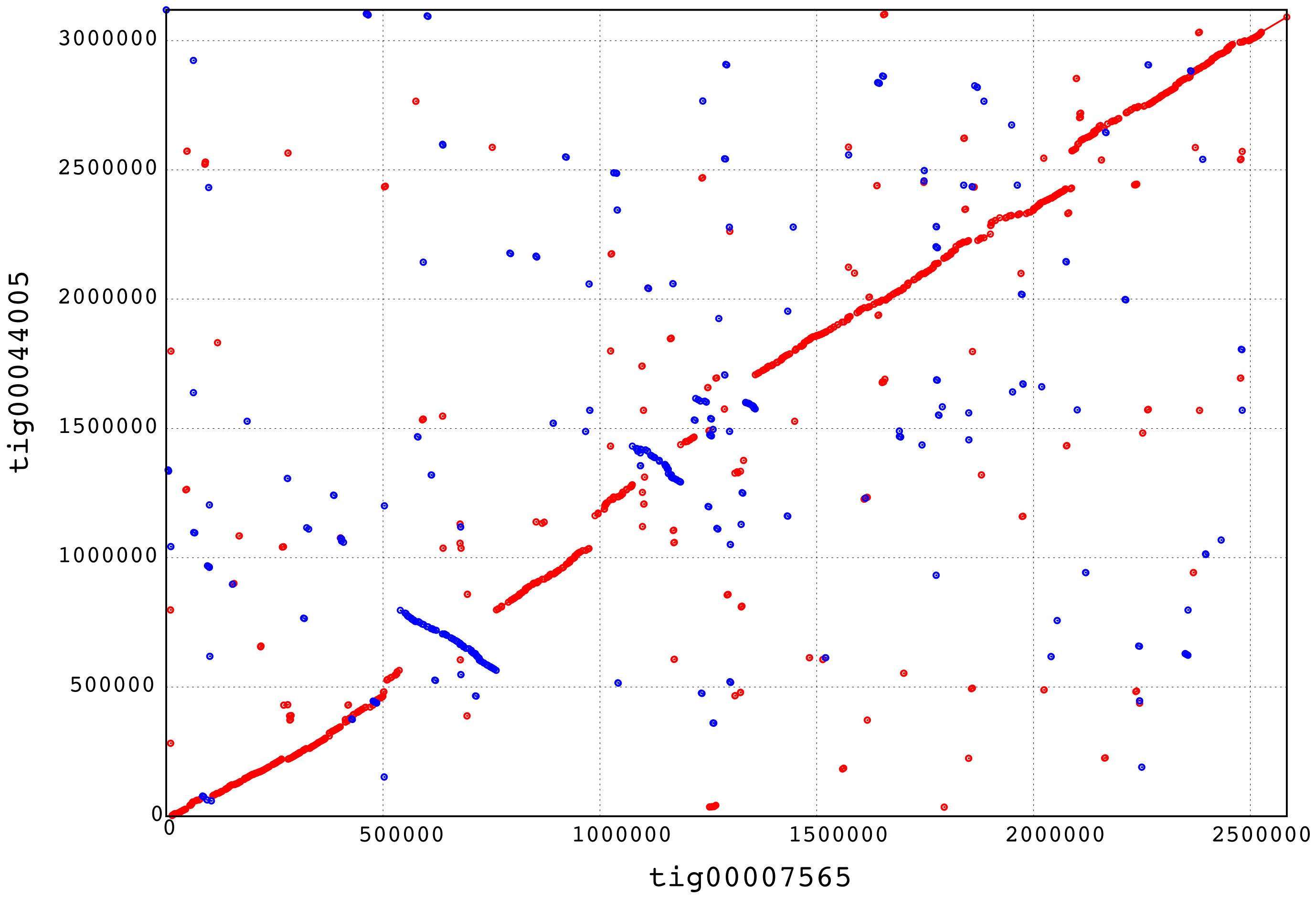

Supplementary Figure 2f. MUMmer dot plot of contig pair #6

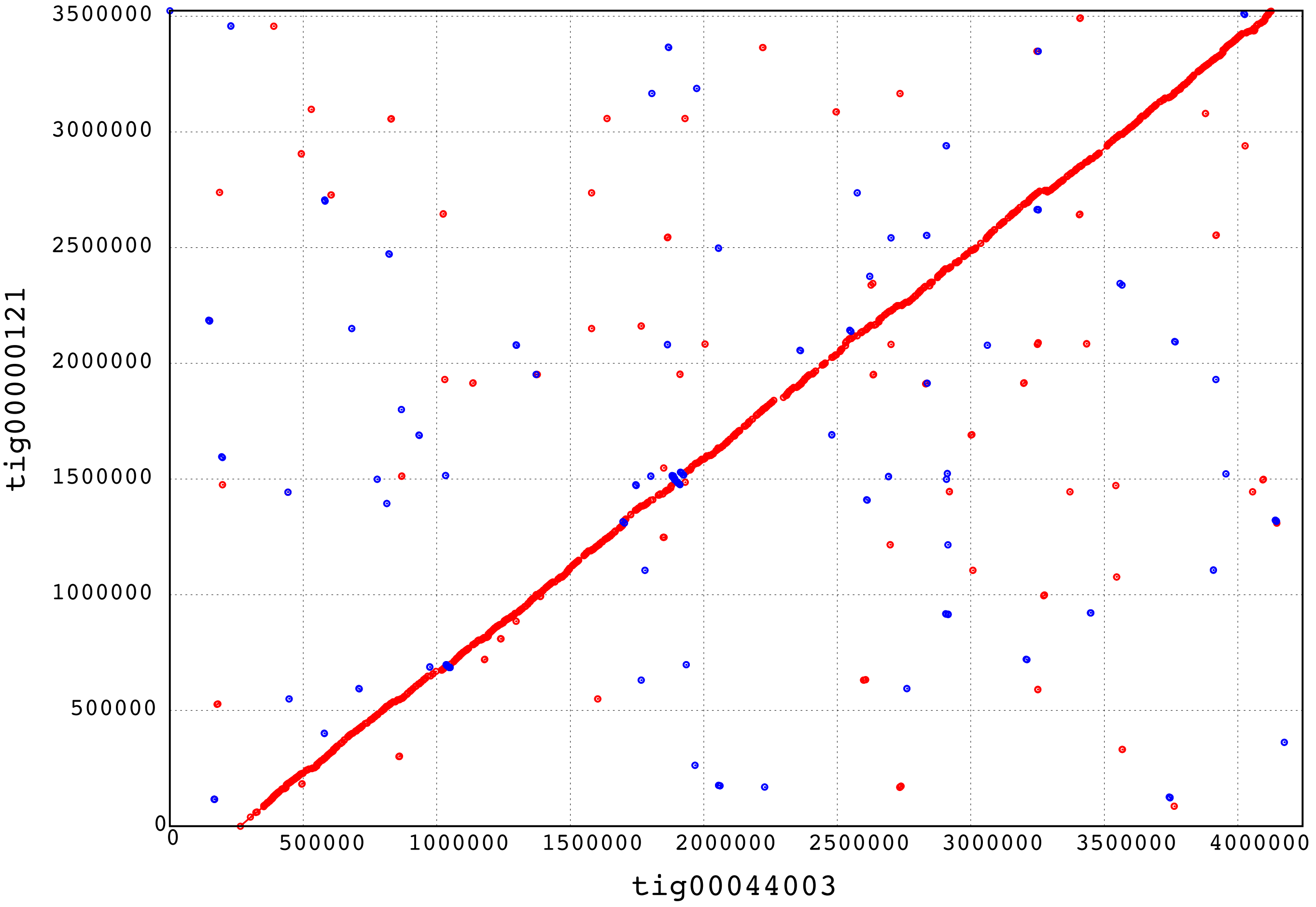

Supplementary Figure 2g. MUMmer dot plot of contig pair #7

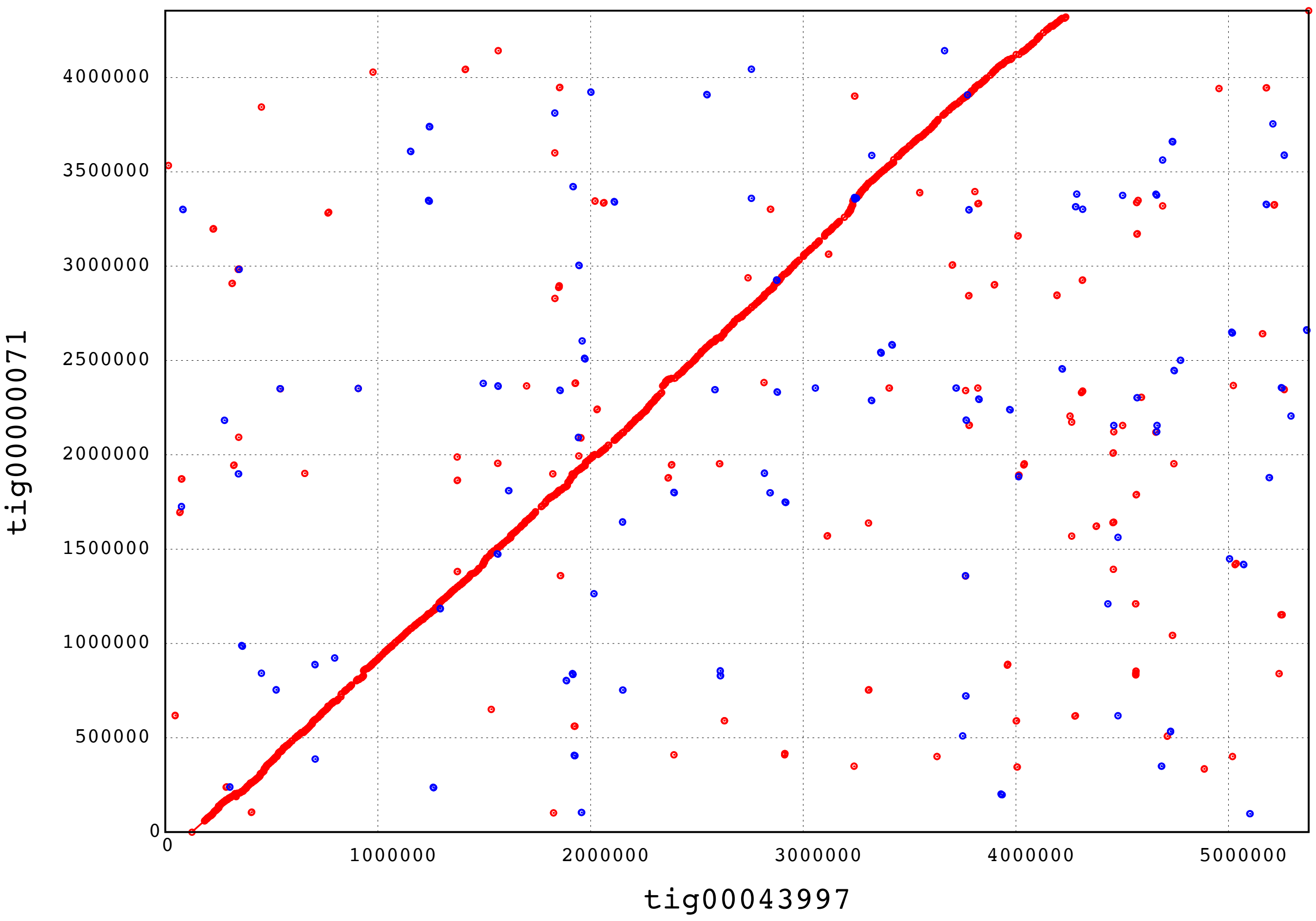

Supplementary Figure 2h. MUMmer dot plot of contig pair #8

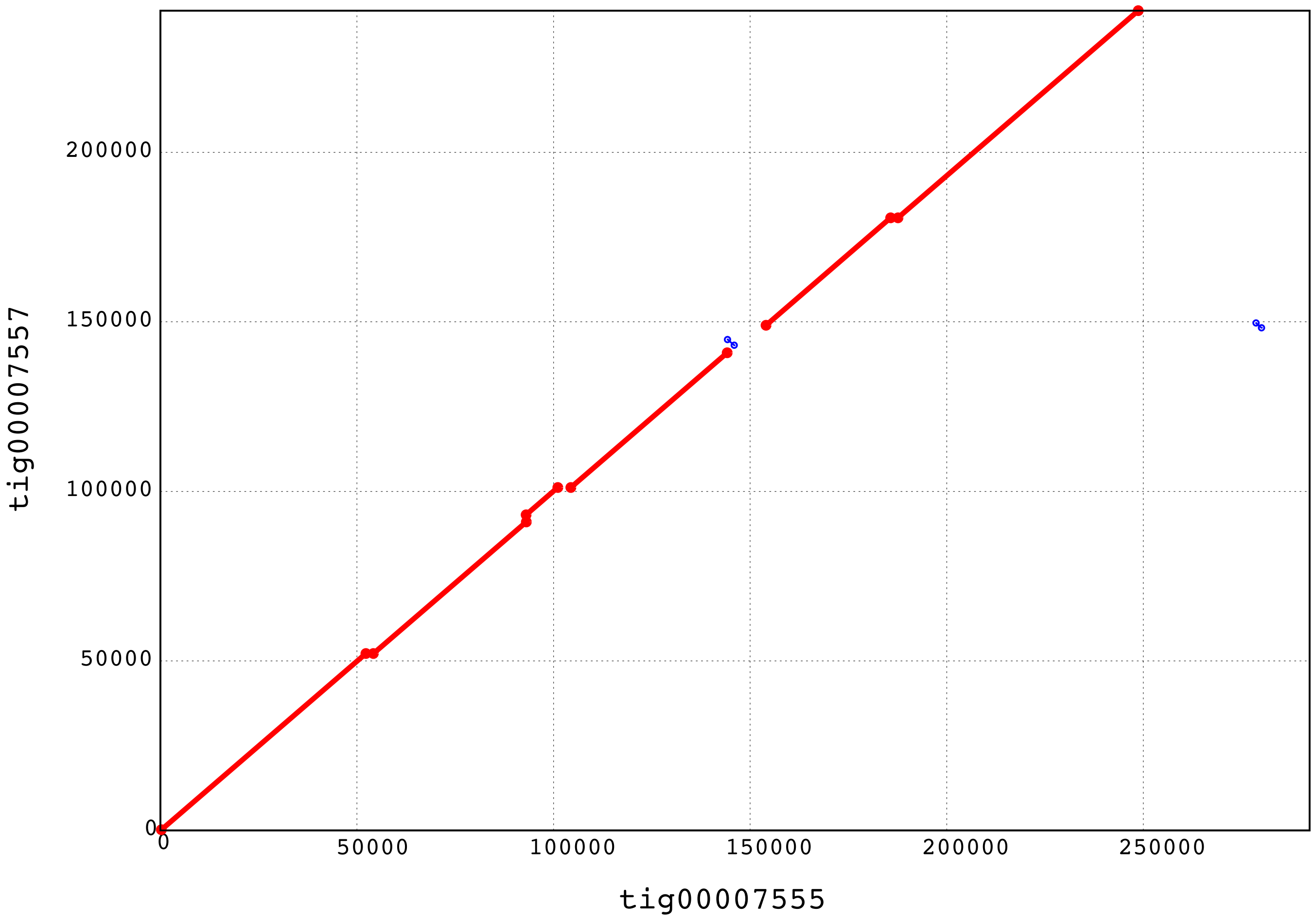

Supplementary Figure 2i. MUMmer dot plot of contig pair #9

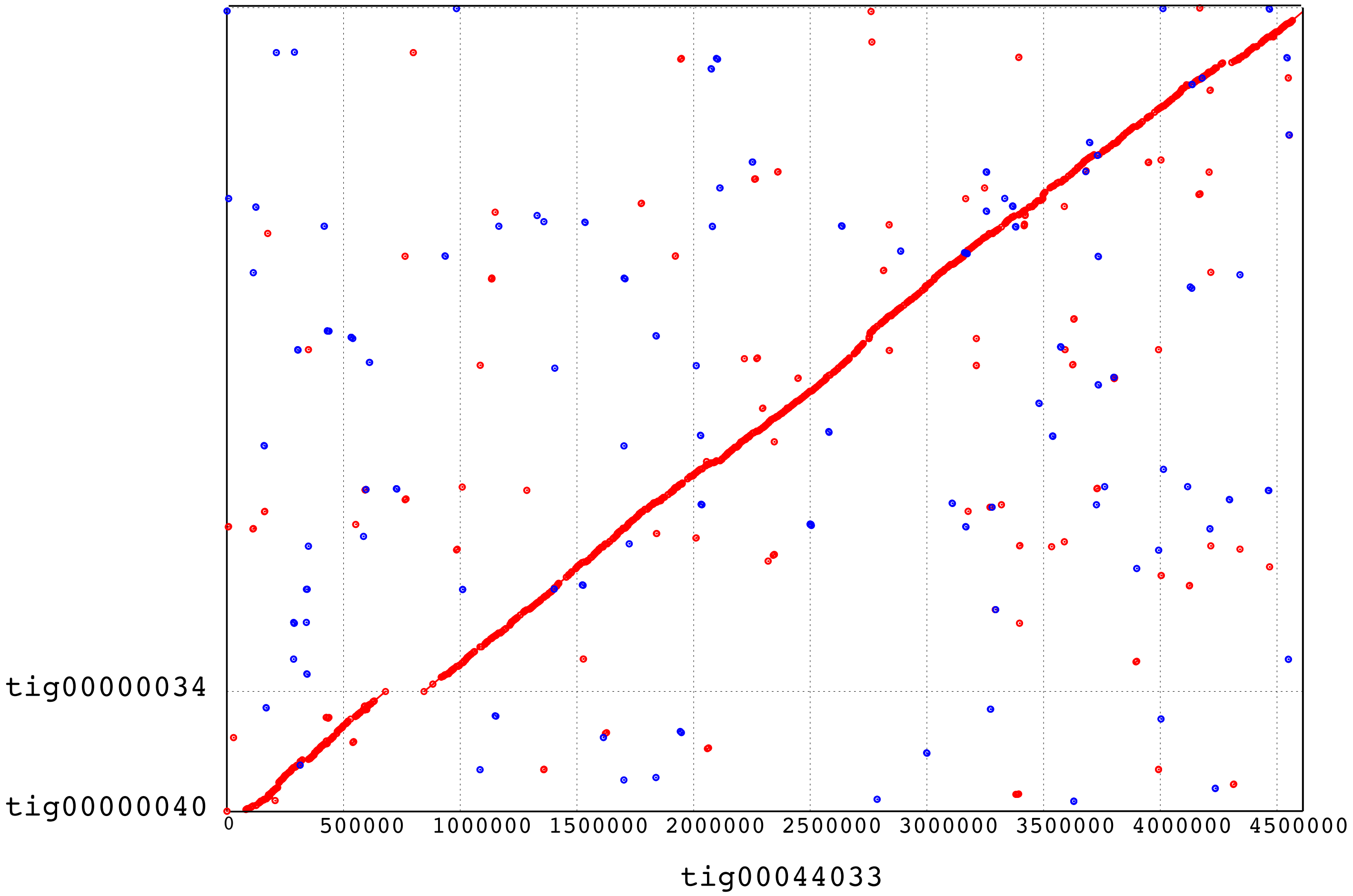

Supplementary Figure 2j. MUMmer dot plot of contig pair #10

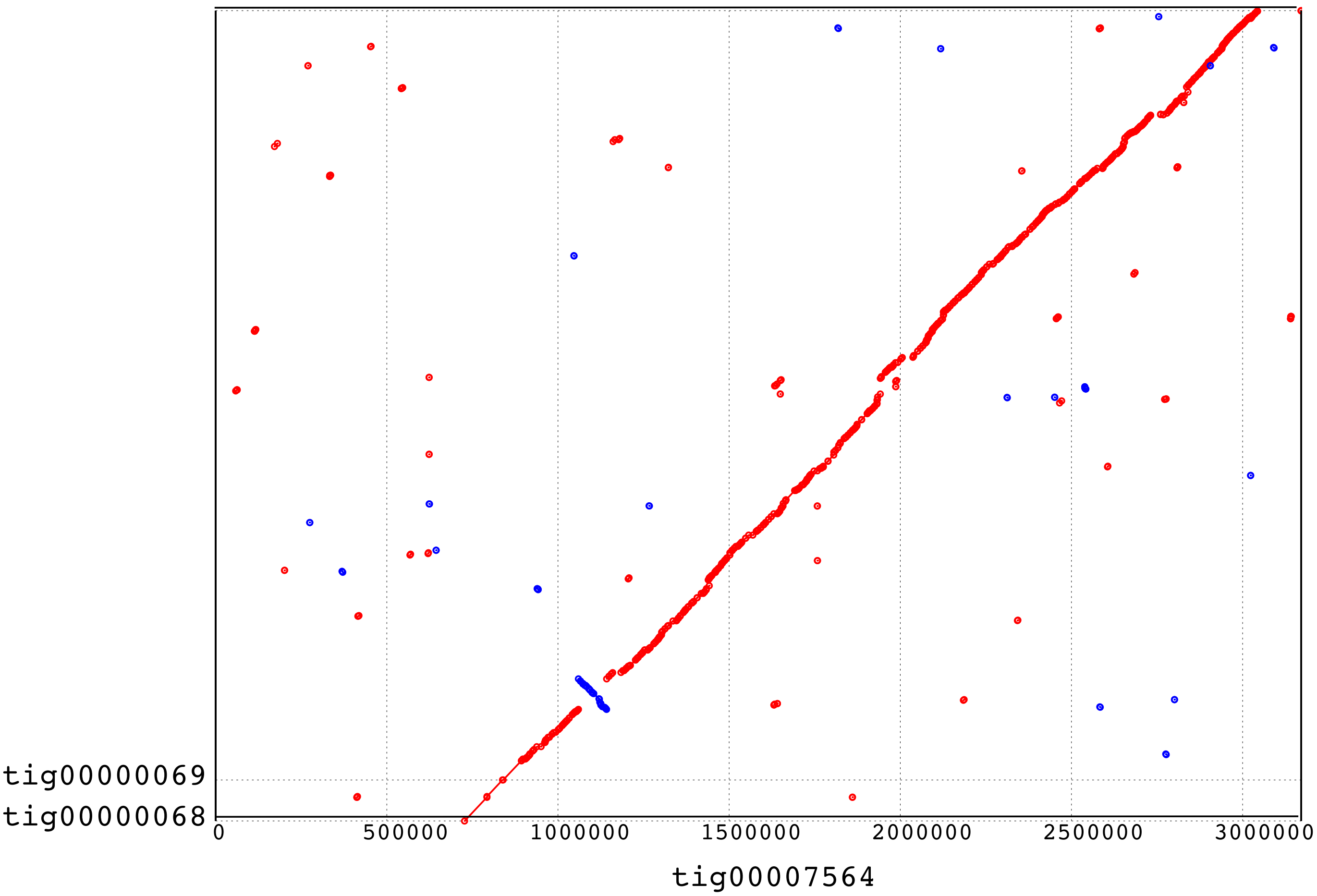

Supplementary Figure 2k. MUMmer dot plot of contig pair #11

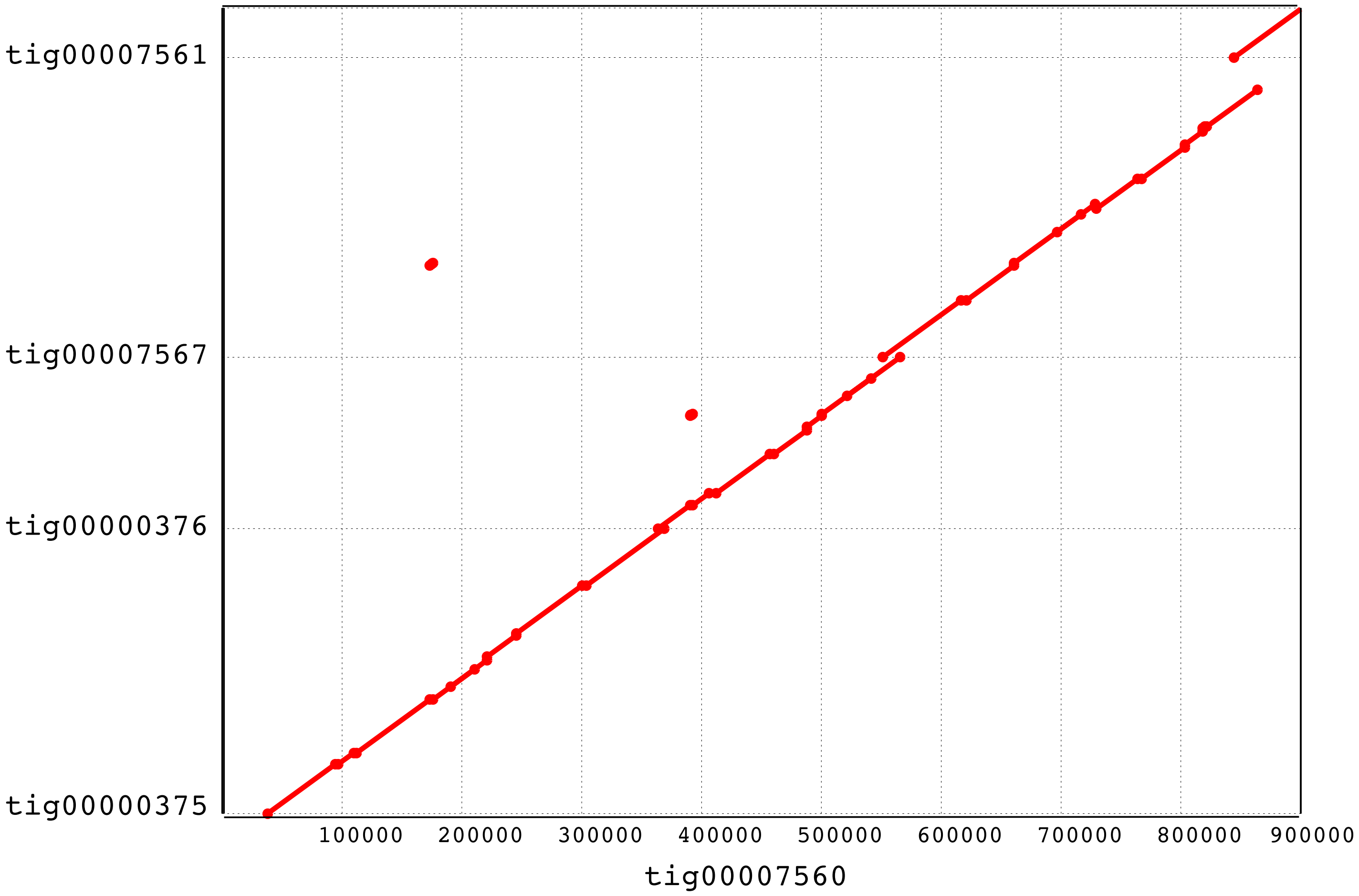

Supplementary Figure 2L. MUMmer dot plot of contig pair #12

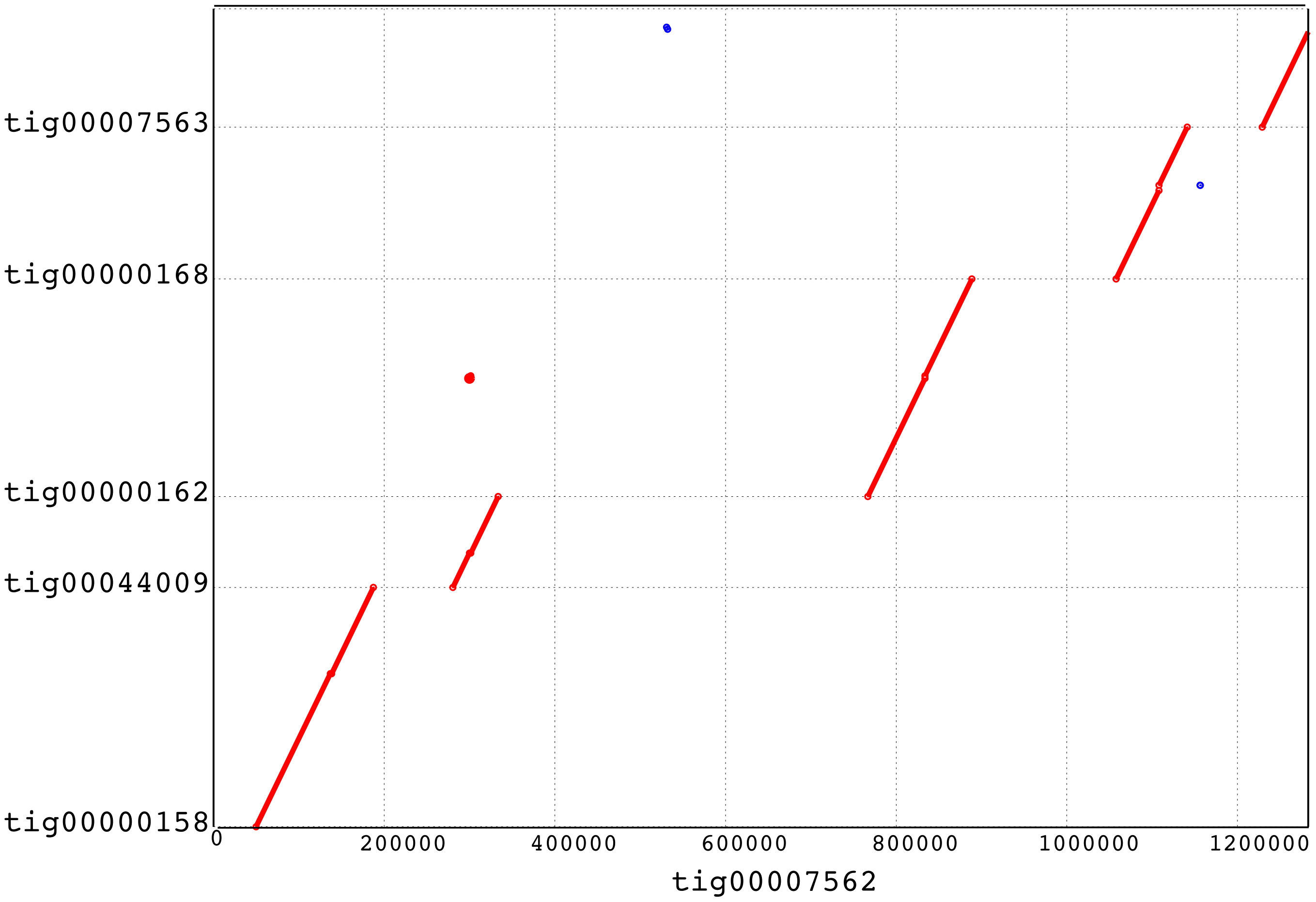

Supplementary Figure 2m. MUMmer dot plot of contig pair #13

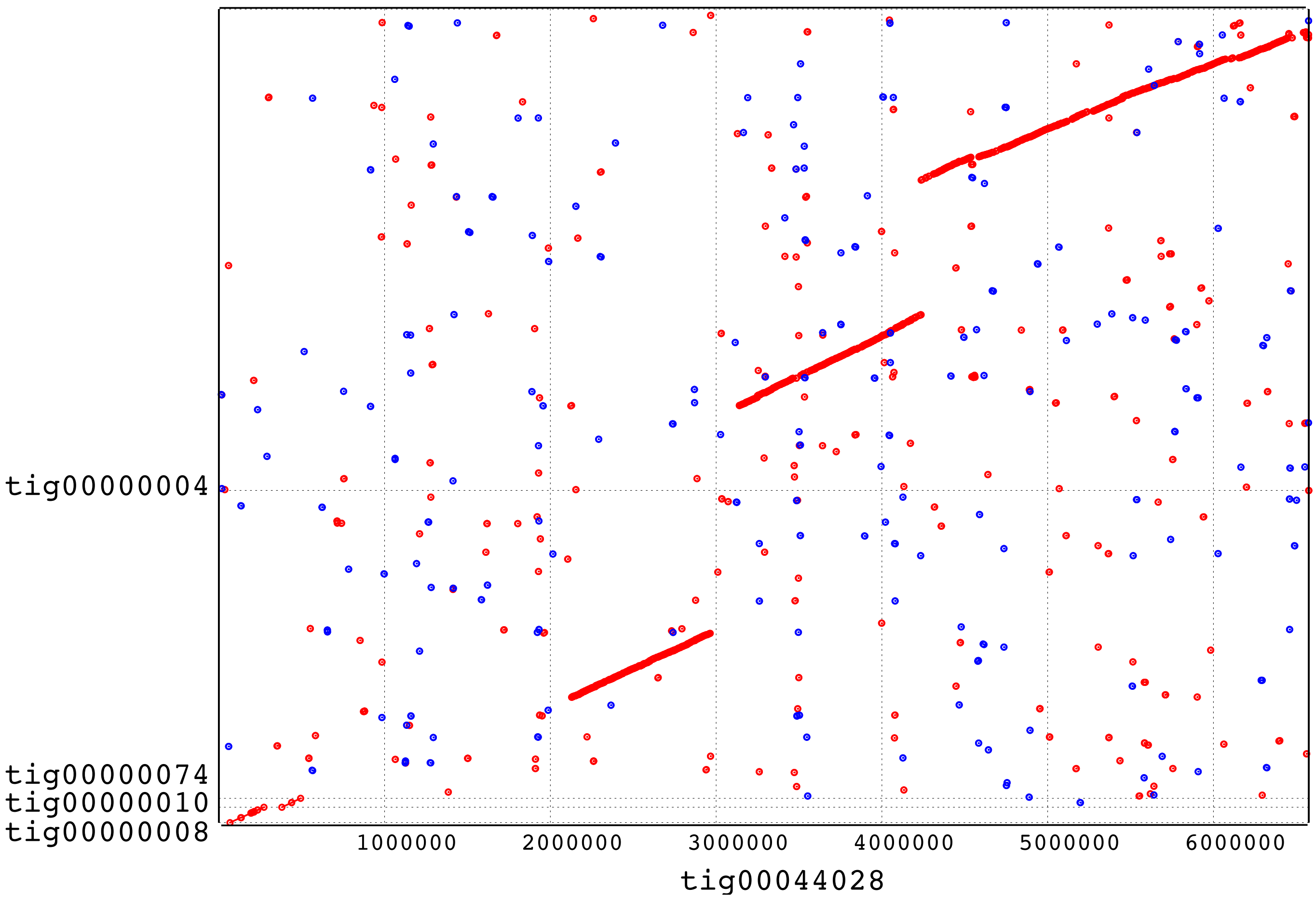

Supplementary Figure 2n. MUMmer dot plot of contig pair #14

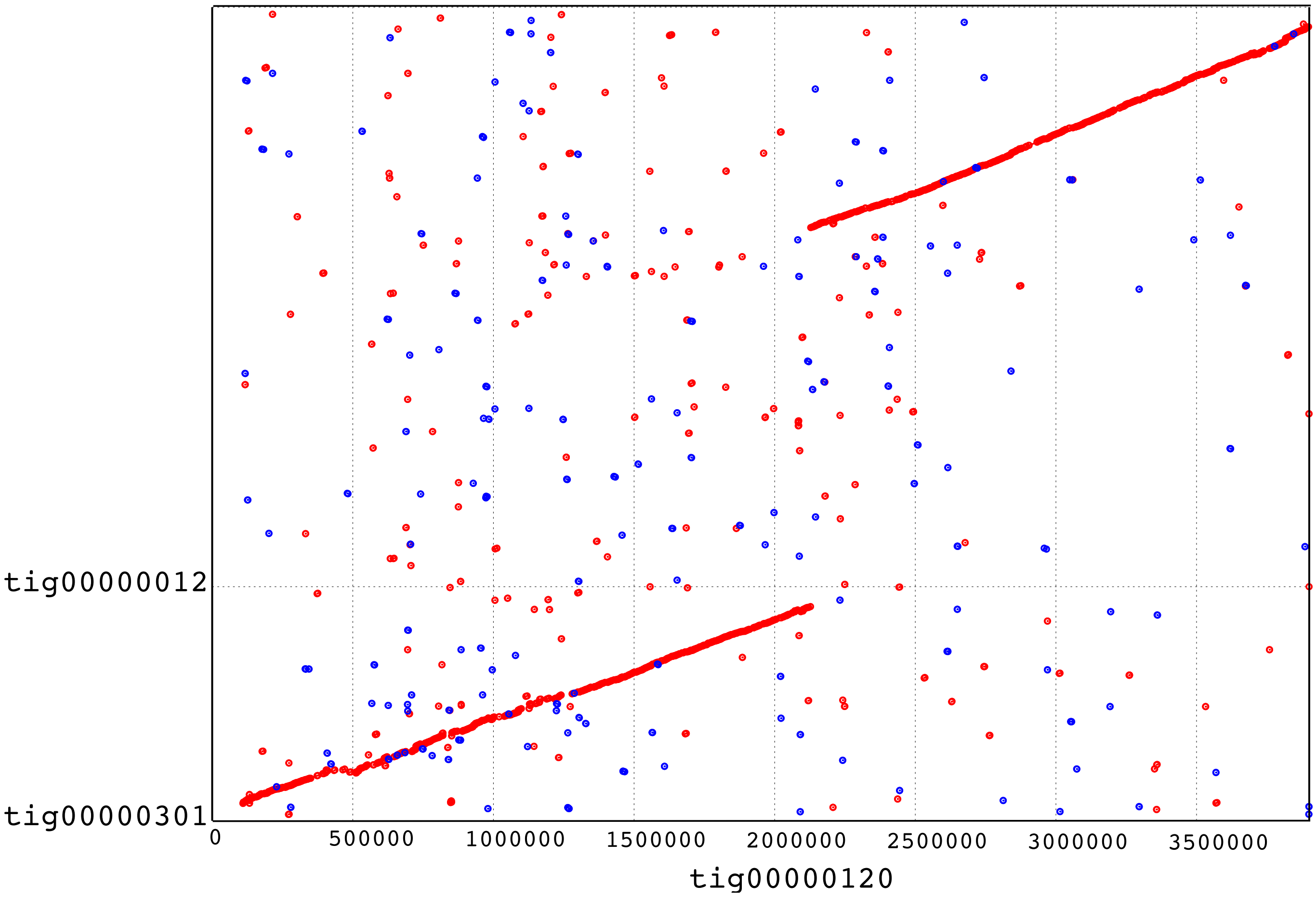

Suppl Figure S3. Transcriptome coverage of predicted coding sequences

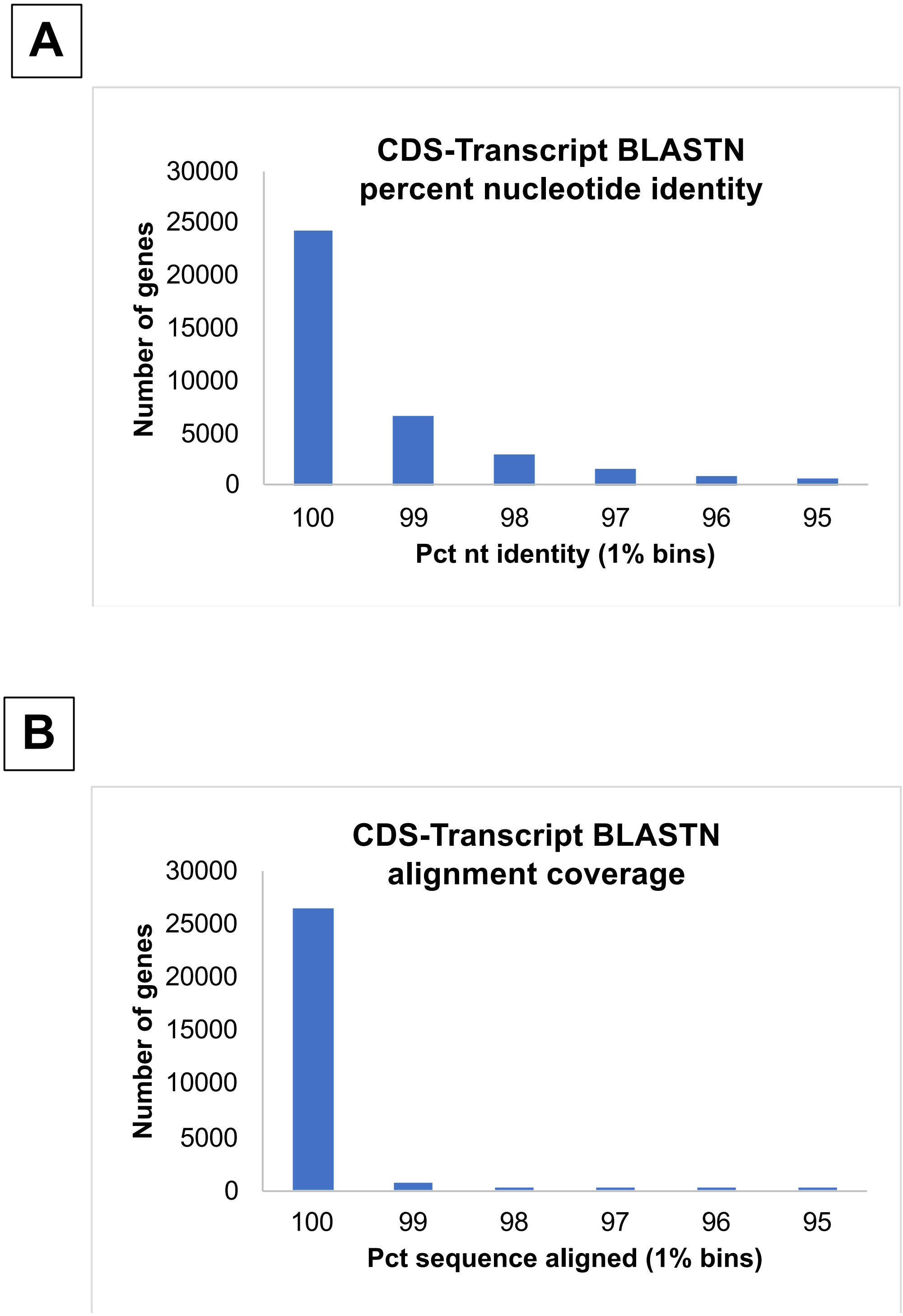

Suppl Figure S4. Sequence heterozygosity in diploid alleles of predicted proteins.

A

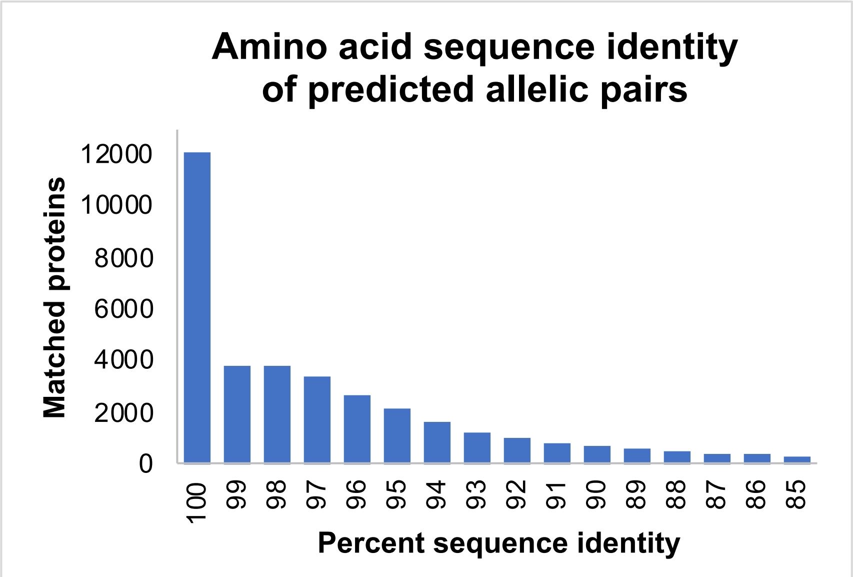

B

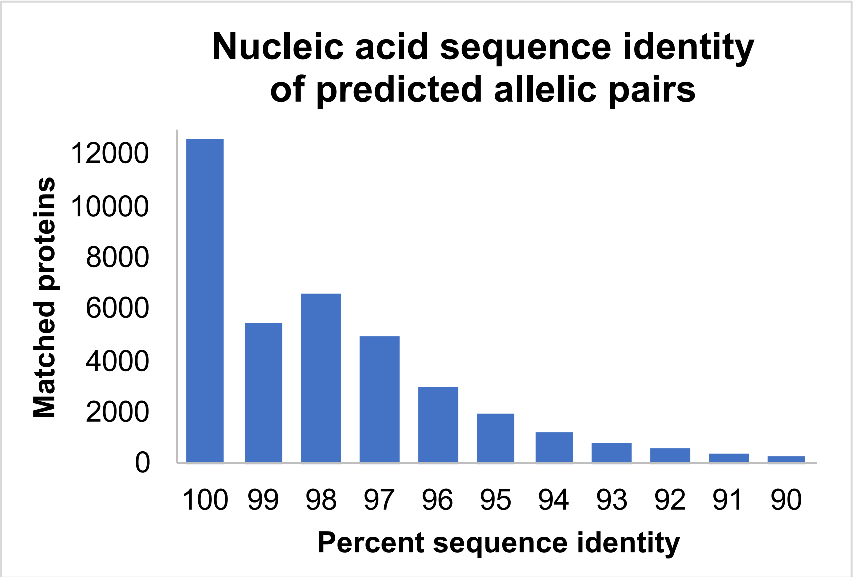

Supplementary Figure S5. Genome-wide heterozygosity based on 17-mer pattern frequency in unassembled sequencing reads. Heterozygosity was estimated from the relative heights of peaks A and B using an empirical formula derived from well-established reference genomes. [2]

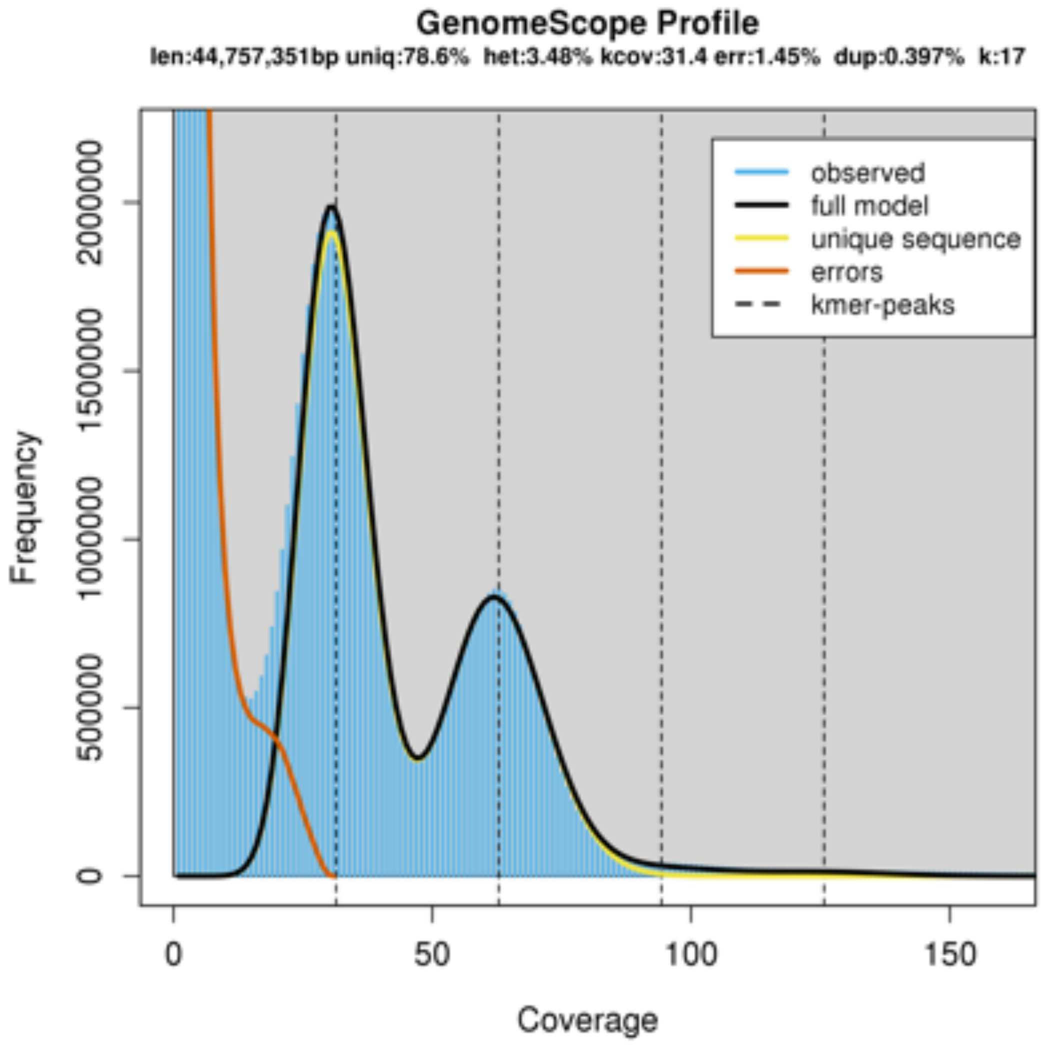

Suppl Figure S6. Telomer repeat patterns. A, Examples from previously sequenced genomes. B, Degenerate repeat patterns from 5' and 3' ends of four *N. inconspicua* contigs with telomer caps at both ends. Red and blue colors mark exact 12-mer repeats at contig leading and trailing ends, respectively, with pattern irregularities shown in black.

A

Phaeodactylum tricornutum

```
>chr_9|1000842|1002813 3' end
GTTAGGGTTAGGGTTAGGGTTAGGGTTAGGGTTAGGGTTAGGGTTAGGGTTAGG
GTTAGGGTTAGGGTTAGGGTTAGGGTTAGGGTTAGGGTTAGGGTTAGGGTTAGG
GTTAGGGTTAGGGTTAGGGTTAGGGTTAGGGTTAGGGTTAGGGTTAGGGTTAGG
GTTAGGGTTAGGGTTAGGGTTAGGGTTAGGGTTAGGGTTAGGGTTAGGGTTAGG
GTTAGGGTTAGGGTTAGGGTTAGGGTTAGGGTTAGGGTTAGGGTTAGGGTGG
```

Thalassiosira pseudonana

```
>chr_22|1056000|1057565 3' end
GTTAGGGTTAGGGTTAGGGTTAGGGTTAGGGTTAGGGTTAGGGTTAGGGTTAGG
GTTAGGGTTAGGGTTAGGGTTAGGGTTAGGGTTAGGGTTAGGGTTAGGGTTAGG
GTTAGGGTTAGGGTTAGGGTTAGGGTTAGGGTTAGGGTTAGGGTTAGGGTTAGG
GTTAGGGTTAGGGTTAGGGTTAGGGTTAGGGTTAGGGTTAGGGTTAGGGTTAGG
GTTAGGGTTAGGGTTAGGGTTAGGGTTAGGGTTAGGGTTAGGGTTAGGGTTAGG
```

Tetrahymena thermophila

```
>GG662216.1 Tetrahymena thermophila scf_8253823 (reverse complement)
TTGGGGTTGGGGTTGGGGTTGGGGTTGGGGTTGGGGTTGGGGTTGGGGTTGGGG
TTGGGGTTGGGGTTGGGGTTGGGGTTGGGGTTGGGGTTGGGGTTGGGGTTGGGG
TTGGGGTTGGGGTTGGGGTTGGGGTTGGGGTTGGGGTTGGGGTTGGGGTTGGGG
TTGGGGTTGGGGTTGGGGTTGGGGTTGGGGTTGGGGTTGGGGTTGGGGTTGGGG
TTGGGGTTGGGGTTGGGGTTGGGGTTGGGGTTGGGGTTGGGGTTGGGGTTGGGG
TTGGGGTTGGGGTTGGGGTTGGGGTTGGGGTTGGGGTTGGGGTTGGGGTTGGGG
TTGGGGTTGGGGTTGGGGTTGGGGT
```

B

```
>tig00044005 len=3118782 reads=5811
AACCCCAACCCTAACCCAACCCTAACCCCAACCCTAACCCTAACCCAACCTAACCCCAAC
CCTAACCCAACCCTAACCCCAACCCTAACCCCAACCCCAACCCTAACCCCAACCCTAACC
CCAACCCTAACCCCAACCCTAACCCTAACCCAACCTAACCCCAACCCTAACCCTAACCCC
AACCCTAACCCTAACCCCAACCTAACCCAACCCTAACCCCAACCCAACCCTAACCCCAAC
CCTAACCCCAACCCTAACCCCAACCCTAACCCCAACCCAAACCCCAACCCTAACCCAACC
CTAACCCCAACCCTAACCTCAACCCTAACCTCAACCCTAACCTCAACCCTAA
...
AGGGTTAGGGTTAGGGTTGGGGTTAGGGTTGGGGTTAGGGTTGGGGTTAGGGTTGGGGTT
AGGGTTGGGGTTAGGGTTGGGGTTAGGGTTGGGGTTAGGGTTAGGGTTGGGGTTAGGGTT
GGGTTAGGGTTGGGGTTAGGGTTAGGGTTGGGTTAGGGTTGGGGTTAGGGTTAGGGTTGG
GGTTGGGGTTGGGGTTTAGGGTTAGGGTTGGGGTTAGGGTTGGGGTTAGGGTTGGGGTTA
GGGTTGGGTTAGGGTTGGGTTAGGGTTGGGGTTAGGGTTGGGGTTAGGGTTGGGGGGTTT
AGGGTTAGGGTTGGGGTTAGGGTTGGGGGTTAGGGTTGGGGTTGGGGGGTTTAGGGTTGG
GGTTAGGGTTGGGGTTGGGTTAGGGTTGGGGTTAGGGTTGGGTTGGG

>tig00000204 len=3108069 reads=5718
CCCAACCCCAACCCTAACCCAACCCTAACCCTAACCCTAACCCCAACCCTAACCCCAACC
CTAACCCAACCCAACCCTAACAACCCTAACCCCAACCCTAACCCAACCCTAACCCAACCC
TAACCCCAACCCTAACCCTAACCCCAACCCTAACCCTAACCCAACCCTAACCCCAACCCT
AACCCCAACCCTAACCCAACCCTAACCCTAACCCCAACCCTAACCAACCCCAACCCTAAC
CCCAACCCTAACCCTAACCCCAACCCTAACCCCAACCCTAACCCCAACCCTAACCCCAAC
CCTAACCCTAACCCCAACCCTAACCCAACCCTAACCCCAACCCTAACCCAACCCTAACCC
CAACCCTAACCCCAACCCTAAC
...
TTAGGGTTGGGGTTAGGGTTGGGGTTAGGGTTGGGGTTAGGGTTGGGGTTAGGGTTAGGG
TTGGGGTTAGGTTGGGGGTTAGGGTTGGGGTTAGGGTTGGGTTAGGGGTTGGGTTAGGGT
CGGGTTTAGGGTTGGGGTTAGGGTTGGGGTTAGGGTTGGGGTTAGGGTTAGGGTTGGGGT
TAGGGTTGGGGTTGGGGTTGGGGTTGGGGTTAGGGTTAGGGTTGGGGTTGGGTTGTTAGG
GTTGTTAGGGTTAGGGTTGGGGTTGGGGTTGGGGTTGGGGTTAGGGTTGGGGTTAGGGTT
GGGGTTAGGGTTGGGGTTTGGGGTTGGGGTTAGGGTTAGGGTTGGG

>tig00000176 len=2972054 reads=5494
CCTAACCCTAACCCTAACCCCAACCCCAACCTAACCCAACCCTAACCCAACCCAACCCTA
ACCCCAACCCTAACCCCAACCCTAACCCAACCCTAACCCTAACACCCCAACCCTAACCCC
AACCCTAACCCCAACCCTAACCCTAACCCCAACCCTAACCCAACCCTAACCCAACCCTAA
CCCCAACCCTAACCCCAACCCTAACCCCAACCCTAACCCTAACCCCAACCCTAACCCCAA
CCCTAACCCAACCCAACCCTAACCCCAACCCCAACCCTAACCCCAACCCTAACCCAACCC
TAACCCCAACCCTAACCCTAACCCCAACCCAACCCAACCCTAACCCTAACCCCAACCCT
...
TTAGGGTTGGGGTTAGGGTTGGGGTTAGGGTTAGGGTTGGGGTTAGGGTTGGGGTTAGGG
TTGGGGTTAGGGTTGGGGTTAGGGTTGGGGTTAGGGTTGGGGTTAGGGTTGGGGTTAGGG
TTGGGGTTGGGGTTAGGGTTGGGGTTAGGGTTGGGGTTAGGGTTGGGGTTAGGTTGGGGT
TAGGGTTGGGGTTAGGGTTGGGTTGGGGTTAGGGTTAGGGTTAGGGTTGGGGGTTAGGGT
TGGGGTTGGTGTTAGGGTTGGGTTAGGGTTAGGGTTAGGGTTAGGGTTGGGGTTAGGGTG
GGGTTGGGGGTTAGGGTTGGGTTAGGGTTGGGGTTAGGGTTGGGGTTGGGGTT

>tig00044023 len=2278761 reads=4113
AACCCTAACCCCAACCCCAACCCTAACCCAACCCTAACCCCAACCCCAACCCTAACCCCA
ACCCTAACCCCAACCCTAACCCCTCAACCCAACCCTAACCCCAACCCTAACCCCAACCCT
AACCCAACCCTAACCCTAACCCCAACCCTAACCCCAACCCCAACCCTAACCCCAACCCTA
ACCCCAACCCTAACCCCAACCCTAACCCCAACCCTAACCCCAACCCCAACCCCAACCCTA
ACCCAACCCTAACCCCAACCCTAACCCAACCCTAACCCTAACCCCAACCCTAACCCTAAC
CCCAACCCCAACCCCAACCCTAACCCTCAACCCTAACCCTAACCCTCAACCCTAACCCCA
ACCCTAACCCTAACCCTAACCCTAA
...
GTTAGGGTTGGGGTTAGGGTTGGGGTTAGGGTTGGGGTTAGGGTTGGGGTTAGGGTTGGGGTTGGGTTGGGG
GTTAGGGTTGGGGTTAGGTTGGGGTTAGGGTTGGGGTTAGGGTTGGGTTAGGGTTGGGGT
TAGGGTTGGGTTAGGGTTGGGTTAGGGTTGGGGTTGGGGTTGGGGGTTGGGGTTAGGGTT
GGGTTAGGGTTGGGGTTAGGGTTGGGGTTAGGGTTGGGGTTAGGGTTGGGGGTTAGGGTT
GGGTTAGGGTTGGGTTAGGGTTGGGGTTAGGGTTGGGGTTAGGGTTGGGGTTAGGGTTGG
GGTTGGGGTTAGGGTTGGGGTTAGGGTTGGGGTTAGGGTTGGGG
```

Suppl Figure S7. Maximum-likelihood phylogeny of putative bicarbonate transporters from diatom genomes. Circles on branches indicate monophyly at the species level, X at the branch tips indicate outgroups. Centric diatom species are colored grey, pennate species are colored black (with the exception of outgroups), and haploid consensus *Nitzschia inconspicua* sequences are indicated in dark red. Id number prefixes refer to Phycocosm genome catalogs for the following diatom species: Phatr2, *Phaeodactylum tricornutum*; Fisso1, *Fistulifera solaris*; Semro1, *Seminavis robusta*; Nithil2, *Nitzschia inconspicua*; Psemu1, *Pseudo-nitzschia multiseries*; Fracy1, *Fragilariopsis cylindrus*; Thaoce1, *Thalassiosira oceanica*; Mintr2, *Minidiscus variabilis*; Thaps3, *Thalassiosira pseudoana*; Cyccr1, *Cyclotella cryptica*.

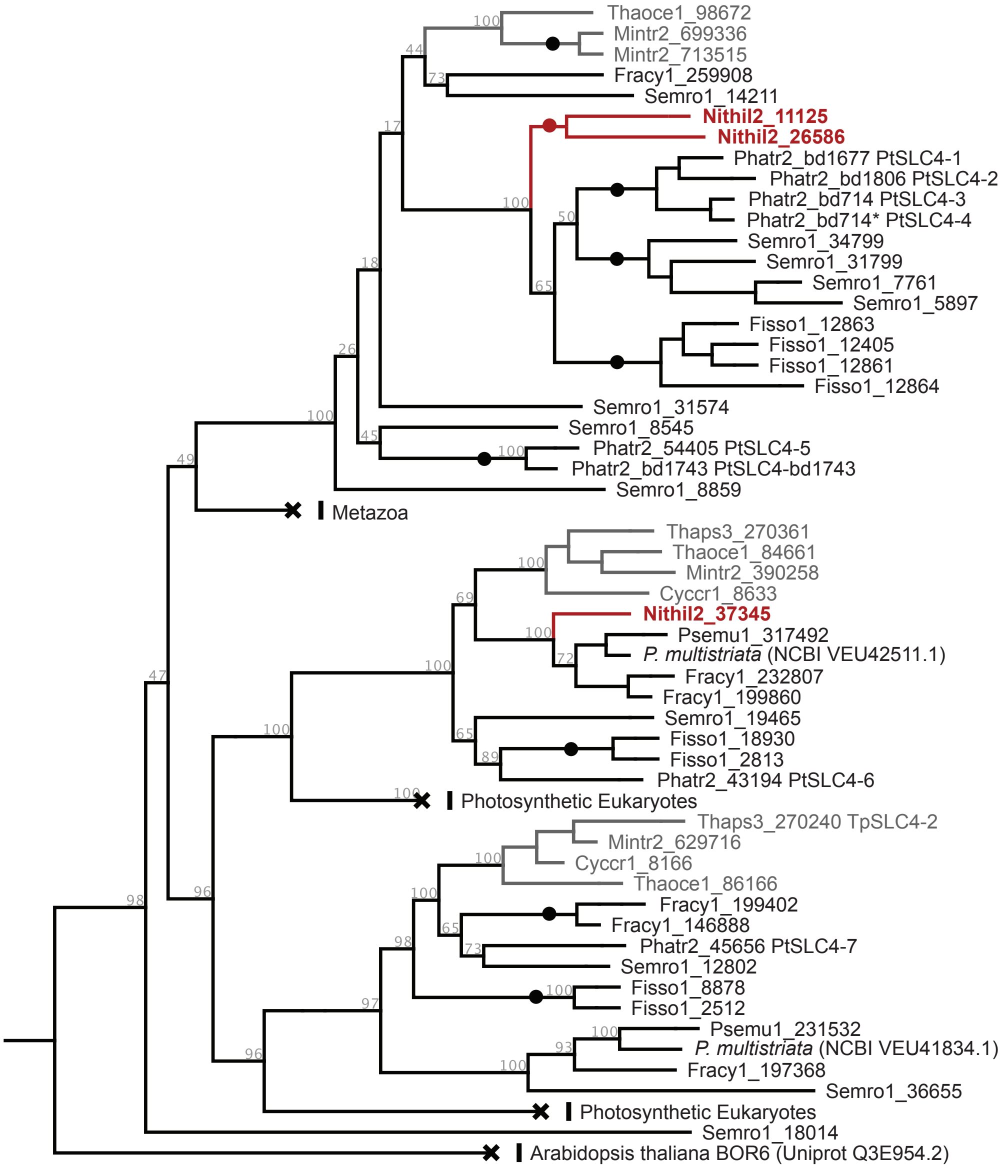

count_cds_partials.pl

```
#!/usr/bin/perl
# count_cds_partials.pl
# Sheila Podell
# March 30, 2013

# takes in a CDS sequence file in fasta format
# creates a tab-delimited table with
# sequence_id, start codon, stop codon

# tallies percentage of CDS that are complete
# meaning they contain expected starts & stops

use warnings;
use strict;

# default values
	my %valid_start_codons = (
	"ATG" => 1,
	"GTG" => 1,
	"TTG" => 1,
	);
	my %valid_stop_codons = (
	"TAA" => 1,
	"TGA" => 1,
	"TAG" => 1,
	);

# get in & out file handles
	unless (@ARGV > 0)
	{
		print "usage: $0 fasta_filename\n";
		exit (0);
	}

# print header
	my @cols = ("cds_file", "num_partial", "num_complete", "total", "pct_complete");
	my $header = join "\t", @cols;
	print "$header\n";

# get data
foreach my $infilename (@ARGV)
{
	open (INFILE, "<$infilename") or die "couldn't open $infilename, $!\n";
	my $flag = 0;
	my @seqs = (); #list of sequence objects
	my $current = "";

# get sequences, check valid start/stop
	my $seqtally = 0;
	my $complete_tally = 0;
	my $partial_tally = 0;

	while (my $line = <INFILE>)
	{
		next if $line =~ /^\s+$/;
		chomp $line;

		if ($line =~ />(.+)/)
```

```
            {
                  if ($flag ==1) #process previous sequence
                  {
                        my $seqstring = $current->{sequence};
                        my $start_codon = substr (uc$seqstring, 0, 3);
                        my $last_3 = (length $seqstring) - 3;
                        my $stop_codon = substr (uc$seqstring, $last_3, 3);
                        $seqtally++;
                        if (exists $valid_start_codons{$start_codon} &&
                              exists $valid_stop_codons{$stop_codon})
                        {
                              $complete_tally++;
                        }
                        else
                        {
                              $partial_tally++;
                        }

                  }

                  my @tmp = split " ", $line;
                  $current = new_seq("record",\@tmp);
                  $flag = 1;
            }
            else
            {
                  $current->{sequence} .= uc"$line";
            }
      }
      close INFILE;
      my $pct_complete = ($complete_tally/$seqtally) *100;
      my $formatted_pct = sprintf ("%.2f", $pct_complete);
      $formatted_pct .= "%";

      my @data = ($infilename, $partial_tally, $complete_tally,$seqtally,$formatted_pct);
      my $output = join "\t", @data;
      print "$output\n";
}

###################################################
# SUBROUTINES
###################################################
sub new_seq {
  my ($className, $param) = @_;
  my $self = {};
  bless $self, $className;
  my @properties = @$param;
  my $header = join " ", @properties;
  $self->{header} = $header;
  $properties[0] =~ s/\>//;
  $self->{id} = $properties[0];

  unless (defined $self->{id})
  {
      warn "no id found for $self->{header}\n";
  }

  $self->{sequence} ="";

  return($self)
}
```

```
sub validate_seq
{
	my ($seqstring) = @_;
	my $seq_length = length ($seqstring);
	my $num_ATCGN = (uc$seqstring =~ tr/ATCGN//);
	unless ($num_ATCGN eq length $seqstring)
	{
		warn "sequence doesn't look like nucleic acid\n";
	}
}
```

References

1 Bohutskyi, P. *et al.* Production of lipid-containing algal-bacterial polyculture in wastewater and biomethanation of lipid extracted residues: Enhancing methane yield through hydrothermal pretreatment and relieving solvent toxicity through co-digestion. *Sci Total Environ* **653**, 1377-1394, doi:10.1016/j.scitotenv.2018.11.026 (2019).

2 Kajitani, R. *et al.* Efficient de novo assembly of highly heterozygous genomes from whole-genome shotgun short reads. *Genome Res* **24**, 1384-1395, doi:10.1101/gr.170720.113 (2014).
